# MAPK14/SLC7A11/GPX4 axis dysregulation drives podocyte ferroptosis via mediating glycerophospholipid metabolism

**DOI:** 10.1038/s41420-026-02990-7

**Published:** 2026-03-11

**Authors:** Shi Qiu, Dandan Xie, Sifan Guo, Zhibo Wang, Ying Cai, Xian Wang, Zhencai Hu, Shiwei Wang, Chunsheng Lin, Hong Yao, Qiang Yang, Yu Guan, Qiqi Zhao, Songqi Tang, Wenjie Sun, Yiqiang Xie, Aihua Zhang

**Affiliations:** 1 School of Chinese Medicine, School of Basic Medical Sciences, International Advanced Functional Omics Platform, Scientific Experiment Center, Hainan Medical University, Haikou, 571199 China; 2 School of Basic Medicine, Graduate School, Second Affiliated Hospital, Heilongjiang University of Chinese Medicine, Harbin, 150040 China; 3 First Affiliated Hospital, Harbin Medical University, Harbin, 150040 China; 4 Department of Nephrology, Traditional Chinese Medicine Integrated Department of Nephrology, The First Affiliated Hospital of Zhengzhou University, Zhengzhou, 450052 Henan China

**Keywords:** Small molecules, Diabetic nephropathy, Mechanisms of disease

## Abstract

Diabetic nephropathy (DN), the leading cause of end-stage renal disease, lacks effective therapies due to an incomplete understanding of its cell-type-specific pathogenesis. Here, through an integrative multi-omics approach, we have decoded the molecular architecture of DN, identify novel therapeutic targets, and validates a promising intervention. Single-cell RNA sequencing of human diabetic kidneys reveals the podocyte as the central cellular nexus of DN, exhibiting specific dysregulation in ferroptosis and glycerophospholipid metabolism, and possessing superior diagnostic potential. High-resolution analysis of podocyte heterogeneity identifies ferroptosis as a key driver of glomerular injury, centered on the dysregulated genes MAPK14/SLC7A11/GPX4. We further demonstrated that astragaloside IV (ASIV) exerts potential protective effects by specifically targeting the ferroptosis pathway, reversing the diabetic transcriptional landscape and preserving podocyte integrity. Spatial metabolomics uncovers profound anatomical compartmentalization of metabolic dysregulation in the renal cortex and medulla, which is effectively regulated by ASIV. Integrated transcriptomic and metabolomic profiling in vitro definitively establishes ferroptosis inhibition as the core mechanism of ASIV-mediated podocyte protection. Finally, clinical metabolomic profiling identifies urinary metabolic intermediates of glycerophospholipid metabolism as highly sensitive and specific non-invasive biomarkers for its diagnosis. Our study delineates a fundamental research framework for DN, from basic mechanism to targeted therapy and precision diagnostics.

## Introduction

Diabetic nephropathy (DN) stands as the leading cause of end-stage renal disease worldwide, posing a formidable challenge to global public health systems [1]. Despite its prevalence, the transition from hyperglycemia to irreversible glomerulosclerosis and renal failure remains incompletely understood, hindering the development of effective disease-modifying therapies. The pathological hallmark of DN is the progressive loss of terminally differentiated glomerular podocytes, a critical event that disrupts the filtration barrier and initiates a cascade of functional decline [2, 3]. While systemic metabolic disturbances are central to DN pathogenesis, the precise cell-type-specific molecular atlas has remained elusive.

Recent advances in single-cell genomics have begun to deconstruct the cellular complexity of the kidney in health and disease [4, 5]. Recent studies have profiled human kidney biopsies, revealing transcriptional shifts in various cell populations during chronic kidney disease [6, 7]. However, these investigations have often been limited by cohort size, resolution, or a focus on advanced fibrosis, leaving a critical gap in our understanding of the cell-specific pathogenic pathways in human DN. In particular, the intrinsic heterogeneity of podocytes and their specific metabolic vulnerabilities have been obscured by tissue analyses, which average signals across diverse cell types [8]. While rodent models have been instrumental in studying DN, the translatability of findings is often questioned due to species-specific differences in renal physiology and disease progression [9, 10]. A systematic, cross-species validation of key pathogenic drivers is therefore essential to bridge this translational gap.

A promising yet underexplored pathway in DN podocytopathy is ferroptosis, an iron-dependent form of regulated cell death characterized by lethal lipid peroxidation [11]. Links between dysregulated cellular metabolism and ferroptosis susceptibility are emerging [12]. In the kidney, perturbations in glutathione metabolism have been implicated in other forms of injury [13, 14], but whether ferroptosis is a principal driver of podocyte loss in human DN, and how it integrates with other metabolic dysregulations, is unknown. This knowledge gap is compounded by a lack of spatial understanding of metabolic alterations within the intricate functional compartments of the kidney, such as the cortex and medulla [15]. Furthermore, the clinical translation of mechanistic insights is hampered by the absence of reliable, non-invasive biomarkers that reflect these underlying molecular pathologies, as current diagnostics like albuminuria and estimated glomerular filtration rate (eGFR) often detect injury only after significant functional impairment has occurred [16].

To address these fundamental challenges, we used a multi-faceted study that integrates cutting-edge single-cell and spatial omics technologies across human patients, murine models, and interventional paradigms. We first constructed a high-resolution, multi-dimensional single-cell atlas of human DN, providing a novel view of the diseased renal ecosystem and nominating the podocyte as the central nexus of pathway dysregulation, with ferroptosis emerging as a top candidate mechanism. We then deepened this insight by decoding podocyte transcriptional heterogeneity directly in human biopsies, unequivocally linking ferroptosis to glomerular injury. To establish conservation and create a platform for therapeutic exploration, we generated a complementary single-cell atlas of the murine diabetic kidney, confirming podocyte-specific metabolic dysregulation as a conserved core driver.

Astragaloside IV (ASIV), a key bioactive saponin from the traditional herb *Astragalus membranaceus*, has long been used for immunomodulation and organ protection. Its documented pharmacological profile includes anti-inflammatory, antioxidant, and anti-apoptotic properties [17, 18]. Previous studies have indicated its renoprotective potential in diabetic models, though its specific role in ferroptosis or lipid metabolism remained unclear. Using a combination of single-cell transcriptomics in vivo and integrated multi-omics in vitro, we demonstrate that ASIV specifically preserves podocyte integrity by inhibiting ferroptosis. To bridge the molecular pathology with tissue-level metabolism, we applied spatial metabolomics to decode the compartmentalized metabolic landscape of DN and its regulation by ASIV, providing a direct metabolic link to its therapeutic efficacy. By systematically linking podocyte ferroptosis to metabolic dysregulation across species and demonstrating its targetability, we illuminate a new axis for therapeutic intervention in this devastating disease. Finally, we translated these mechanistic findings to clinical fields by identifying and validating a panel of urinary metabolites as highly sensitive and specific non-invasive biomarkers for DN diagnosis, directly connecting our molecular discoveries to potential clinical applications.

## Result

### Multi-dimensional single-cell atlas decodes podocyte-specific pathway dysregulation

**Fig. 1:**
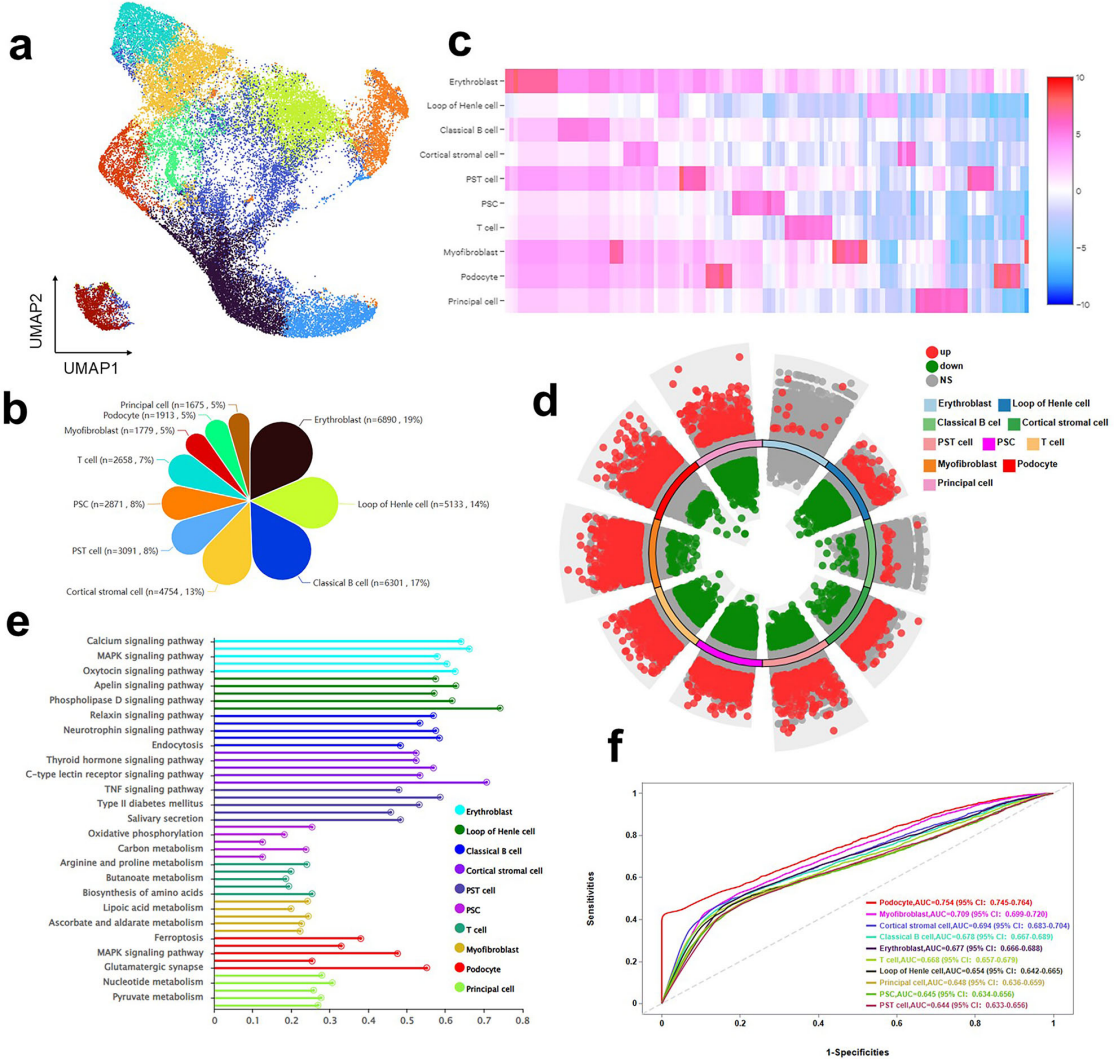
Single-cell transcriptomic atlas reveals cellular heterogeneity and molecular alterations in human diabetic kidney. **a** Global cellular landscape of human DN kidneys. Uniform Manifold Approximation and Projection (UMAP) visualization of 37,065 high-quality nuclei isolated from renal biopsies of DN patients (*n* = 3). Cells are color-coded by their identity across 10 major cell types: Erythroblast, Loop of Henle cell, Classical B cell, Cortical stromal cell, Proximal straight tubular cell (PST cell), Proximal segment cell (PSC), T cell, Myofibroblast, Podocyte, and Principal cell. The integrated analysis shows both distinct clustering of cell types and shifts in population structure between conditions. **b** Quantitative composition of the DN renal cells. The petal pie chart illustrates the proportional abundance of each major cell type, expressed as a percentage of the total cells analyzed across all DN samples. **c** Distribution status of each major cell type (Erythroblast, Loop of Henle cell, Classical B cell, Cortical stromal cell, Proximal straight tubular cell (PST cell), Proximal segment cell (PSC), T cell, Myofibroblast, Podocyte, and Principal cell). **d** Volcano plot summarizing differential gene expression. The plot displays all differentially expressed genes (DEGs) across all major cell types combined in DN. Significantly upregulated (red) and downregulated (green) genes are highlighted. **e** Functional pathway enrichment analysis. Bar graph showing the top 5 significantly enriched KEGG pathways (*y*-axis) for each major cell type in DN, based on hypergeometric testing of regulated DEGs. Pathways are color-coded by cell type. Critical DN-associated pathways are identified, including ferroptosis, glycerophospholipid metabolism, MAPK signaling pathway, glutathione metabolism, and glutamatergic synapse in podocytes. **f** Diagnostic potential of each cell population. ROC curves evaluating the performance on the AUC value of each major cell type’s transcriptional signatures.

We present a comprehensive cellular atlas of human DN by performing single-nucleus RNA sequencing on 37,065 high-quality nuclei from patient renal biopsies. Subsequently, Uniform Manifold Approximation and Projection visualization delineates the renal ecosystem across ten major cell types—Erythroblast, Loop of Henle cell, Classical B cell, Cortical stromal cell, Proximal straight tubular cell (PST), Proximal segment cell (PSC), T cell, Myofibroblast, Podocyte, and Principal cell. This analysis reveals not only distinct, identity-based clustering but also critical, disease-associated shifts in cellular population structure that illuminate the complex pathobiology of DN (Fig. 1a). Furthermore, quantitative analysis of the cellular composition, depicted in a petal pie chart, precisely delineates the proportional abundance of each major cell type, thereby establishing a critical baseline and revealing potential pathogenic alterations in the renal cellular ecosystem underlying DN progression (Fig. 1b). Based on this, analysis of the distribution status of all ten major renal cell types within the DN landscape reveals critical relationships and specific alterations (Fig. 1c). To assess transcriptional dysregulation, a genome-wide volcano plot summarizes all differentially expressed genes (DEGs) across the combined renal cell types, highlighting significantly upregulated (red) and downregulated (green) genes, which reveals the pervasive nature of the disease and pinpoints key molecules (Fig. 1d). Moreover, functional pathway enrichment analysis, illustrated as a bar graph of the top five significantly enriched KEGG pathways for each cell type, uncovers cell-type-specific pathway dysregulation. Notably, podocytes exhibit critical alterations in ferroptosis, glycerophospholipid metabolism, and glutamatergic synapse, thereby pinpointing novel mechanistic underpinnings of glomerular injury and revealing potential cell-targeted therapeutic strategies for DN (Fig. 1e and Table S2). Importantly, evaluation of the diagnostic potential for each major renal cell population using ROC curves reveals that podocytes exhibit superior discriminatory power with the highest AUC value of 0.754, significantly outperforming other cell types, thereby considering podocyte-specific gene expression patterns as a particularly sensitive and promising biomarker for distinguishing DN (Fig. 1f). Together, this multi-dimensional single-cell atlas decodes podocyte-specific pathway dysregulation, highlighting ferroptosis, glycerophospholipid metabolism, and glutamatergic synapse as central mechanisms—and establishes podocytes as the cellular nexus of disease pathogenesis, thereby providing a critical foundational resource for understanding DN pathobiology and illuminating cell-targeted therapeutic avenues for this complex disease.

### Decoding podocyte heterogeneity identifies ferroptosis as a key driver of glomerular injury

**Fig. 2:**
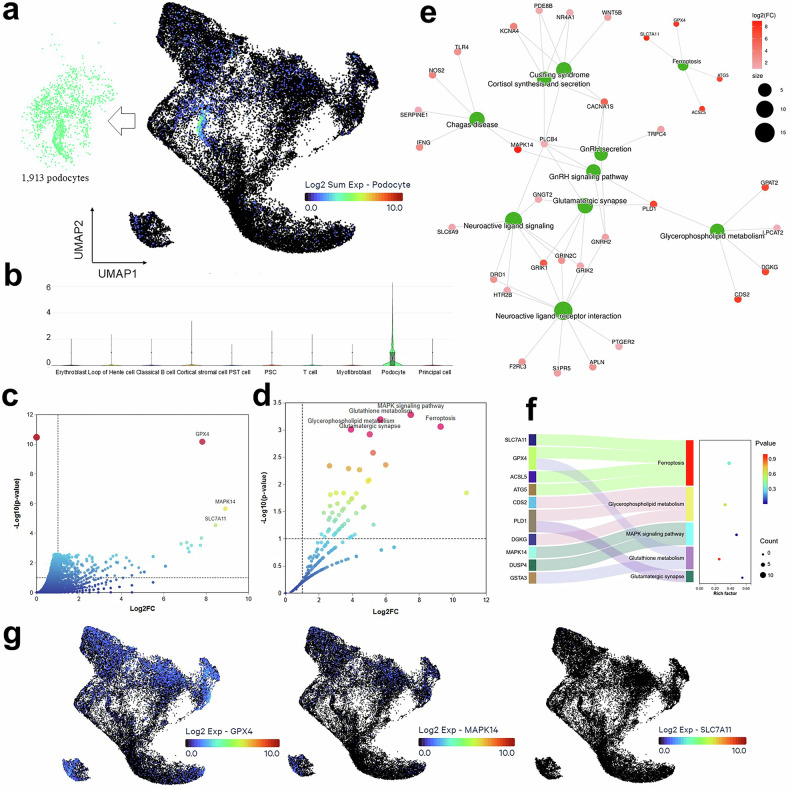
Single-cell profiling unveils podocyte heterogeneity and implicates ferroptosis. **a** Identification of podocyte populations human DN kidneys. UMAP visualization of 1,913 podocytes extracted from the integrated single-cell dataset in DN samples. **b** Validation of podocyte identity based on the classical markers. Violin plots display the normalized expression levels of canonical podocyte markers. The plots confirm cell identity and reveal heterogeneous expression patterns defining distinct podocyte states. **c** Differential gene expression profile of DN podocytes. Volcano plot displaying all genes analyzed from podocytes in DN samples (Log2 fold-change on the *x*-axis; -Log10 adjusted *p* value on the *y*-axis). Significantly regulated genes are highlighted, with key genes annotated. **d** Pathway enrichment analysis of podocyte DEGs and the top significantly enriched KEGG pathways based on podocyte-specific DEGs. Key pathways related to DN pathogenesis are labeled, including ferroptosis, glycerophospholipid metabolism, MAPK signaling pathway, glutathione metabolism, and glutamatergic synapse. The -Log10 (adjusted *p* value) indicating the enrichment significance. **e** Integrated gene-pathway enrichment network. The network visualization connecting differentially expressed genes to their significantly enriched KEGG pathways. Lines represent gene–pathway associations, illustrating how multiple coordinated genes drive the enrichment of specific pathogenic processes, with ferroptosis and glycerophospholipid metabolism emerging as key central mechanism. **f** Sankey Bubble Chart mapping genes to biological pathways. The chart provides a qualitative overview of the relationship between DEGs (left) and enriched pathways (right). The width of the Sankey flows corresponds to the number of genes associated with a pathway, and the bubble size on the right represents the pathway enrichment significance. This highlights the most contributive ferroptosis pathways in podocyte injury. **g** The expression patterns of key regulatory genes. UMAP plots showing the expression distribution of critical genes (GPX4, MAPK14, SLC7A11) involved in the ferroptosis and MAPK signaling pathways across the podocyte cluster.

To decode podocyte heterogeneity and identify the key drivers of glomerular injury in human DN, we performed a high-resolution analysis of 1913 podocytes from patient biopsies. Subsequently, a UMAP visualization, extracted from the global cellular atlas, effectively captured the transcriptional landscape of these cells (Fig. 2a). Furthermore, violin plots validating podocyte identity through canonical marker expression not only confirmed successful isolation but, importantly, revealed significant heterogeneity in the expression of these genes, thereby uncovering distinct transcriptional states within the podocyte compartment (Fig. 2b). Subsequently, a volcano plot of the differential gene expression profile visualized the widespread transcriptomic dysregulation and highlighted key significantly regulated genes, including MAPK14, SLC7A11, and GPX4, as top candidates for further investigation (Fig. 2c and Table S3). Consequently, pathway enrichment analysis of podocyte-specific DEGs confirmed significant enrichment in critical pathways such as ferroptosis, MAPK signaling, glutathione metabolism, glycerophospholipid metabolism, and glutamatergic synapse, thereby providing a mechanistic framework for understanding podocyte loss (Fig. 2d and Table S4). To systematically investigate the molecular underpinnings, PPI network illustrated strong interactions among proteins encoded by dysregulated genes in DN podocytes. To integrate these findings, we constructed a gene-pathway network that directly links DEGs to their enriched biological pathways, unambiguously identifying ferroptosis and glycerophospholipid metabolism as pivotal, centrally connected mechanisms (Fig. 2e). Moreover, a Sankey Bubble Chart mapping genes to pathways identified ferroptosis as the most significant mechanism in podocyte injury, visually emphasizing the important contribution of key genes like SLC7A11, GPX4, and MAPK14 (Fig. 2f). Finally, UMAP plots displaying the expression patterns of these key regulatory genes visualized their distribution and heterogeneity at single-cell resolution (Fig. 2g). In summary, this high-resolution decoding fundamentally advances our understanding of DN by establishing ferroptosis as a central driver of glomerular injury, directly linking podocyte transcriptional states to a specific, therapeutically targetable cell death pathway and providing a precise molecular framework centered on key regulators that explains podocyte loss and opens new avenues for podocyte-protective therapies.

### Murine kidney atlas identifies a podocyte-specific metabolic dysregulation as a core disease driver

**Fig. 3:**
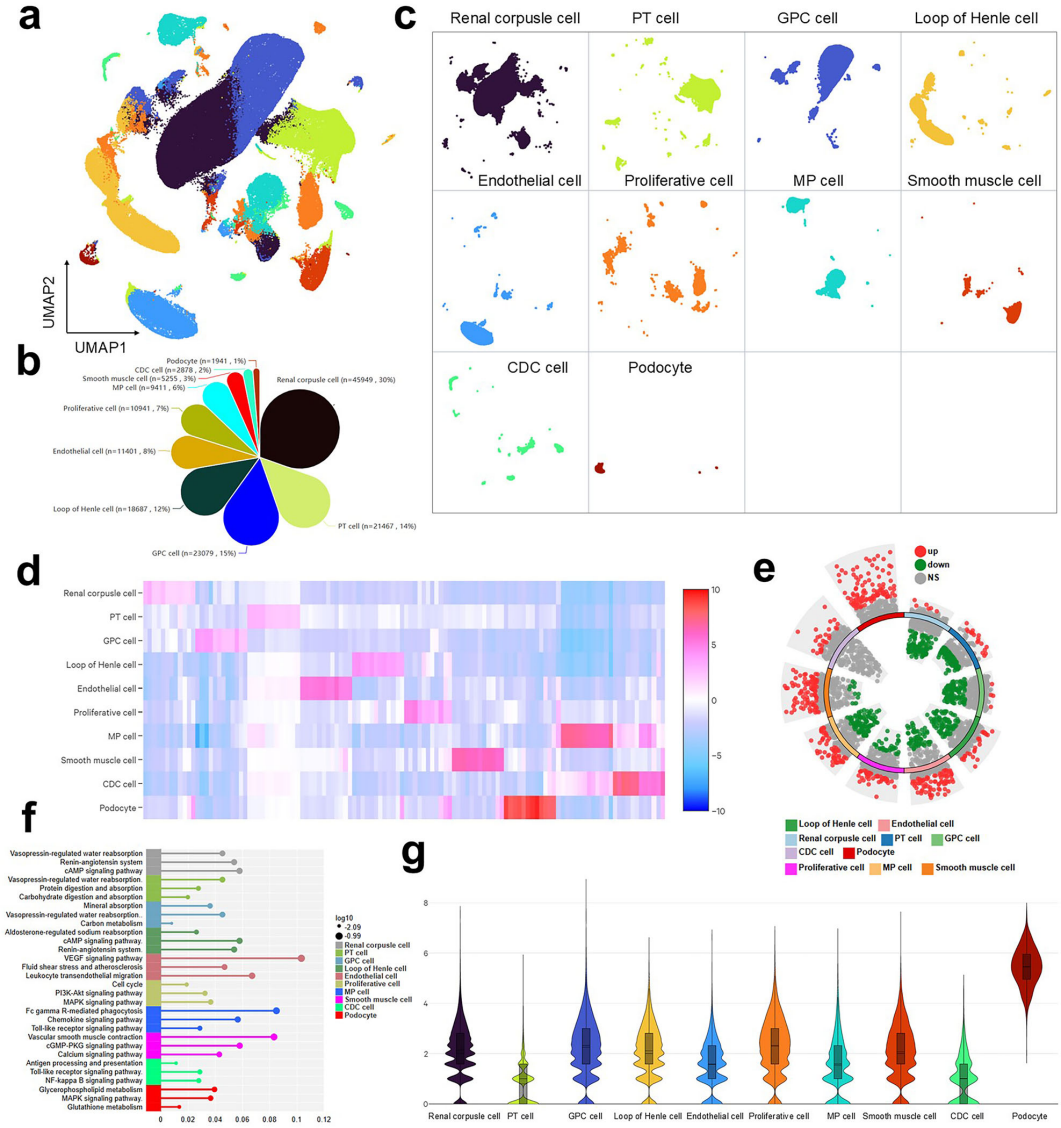
Single-cell transcriptomic profiling of the db/db mouse model reveals conserved cellular alterations. **a** Comprehensive atlas of renal cell types in diabetic and control mice. UMAP visualization of 151,009 high-quality cells obtained from renal tissues of diabetic (db/db, *n* = 3) and non-diabetic control (db/m, *n* = 3) mice. Single-cell suspensions were processed using the 10× Genomics platform. Cells are color-coded by their identity across 10 major renal cell types: Renal corpuscle cell, Proximal tubular cell (PT cell), General proximal cell (GPC cell), Loop of Henle cell, Endothelial cell, Proliferative cell, Mononuclear phagocyte (MP cell), Smooth muscle cell, Conventional dendritic cell (CDC cell), and Podocyte. **b** Quantitative shifts in renal cellular composition. Petal pie chart comparing the proportional abundance of each major cell type expressed as a percentage of total cells. **c** High-resolution clustering reveals novel cellular subpopulations. UMAP visualization of re-clustered cells within each major cell type states. **d** Cell-type-specific transcriptional alterations in diabetes mice kidney. Heatmap displaying the top significantly differentially expressed genes (columns) for each major cell type (rows) in db/db compared to db/m mice. Z-scores of normalized expression demonstrate both conserved and cell-type-specific responses to diabetic states. **e** Genome-wide differential gene expression profile. Volcano plot visualizing all differentially expressed genes in db/db versus db/m mice across all cell types. Significantly upregulated (red) and downregulated (green) genes are highlighted. **f** Pathway-level consequences of transcriptional alterations in each cell type. Bar graph showing the top 3 significantly enriched KEGG pathways based on genes differentially expressed in db/db kidneys. Critical pathways, including glycerophospholipid metabolism, MAPK signaling pathway, and glutathione metabolism are prominently enriched in podocytes, revealing systemic metabolic dysregulation. **g** Violin plots showing expression of major cell type identity based on the canonical marker genes used for cell type annotation.

Consistent with the diabetic phenotype, db/db mice exhibited significantly increased body weight, hyperglycemia, and elevated HbA1c compared to db/m controls (*p* < 0.0001; Fig. S1), confirmed DN progression. To identify conserved pathogenic drivers of DN, we constructed a comprehensive single-cell atlas of the murine diabetic kidney; consequently, it revealed podocyte-specific metabolic dysregulation as a core disease mechanism. This atlas was established by performing UMAP visualization on 151,009 high-quality cells from diabetic (db/db) and non-diabetic (db/m) control mice, thereby successfully delineating the renal microenvironment across ten major cell types, including podocytes and proximal tubular cells, which provides a foundational resource for identifying conserved cellular alterations in DN (Fig. 3a). Subsequently, a petal pie chart quantifies the proportional abundance of each cell type, offering a critical baseline for comparing cellular composition between diabetic and control mice (Fig. 3b). Furthermore, high-resolution re-clustering within these major types, visualized via UMAP, unveils previously unappreciated cellular populations and distinct transcriptional states, indicating a greater complexity of the renal system (Fig. 3c). To delineate the landscape of transcriptional alterations, a heatmap displays the top significantly DEGs for each cell type in diabetic mice, where heatmap distinguish potential targets for therapeutic intervention (Fig. 3d). Moreover, genome-wide profiling of differential gene expression across all cell types reveals a profound molecular landscape, with a volcano plot highlighting widespread significant upregulation and downregulation of genes at tissue-level (Fig. 3e). Critically, we identify core pathway-level disruptions, where enrichment analysis locates glycerophospholipid metabolism, MAPK signaling, and glutathione metabolism as the top significantly altered pathways in podocytes, thus defining the specific metabolic dysregulation that compromises their function (Fig. 3f and Table S5). Additionally, validation using canonical marker genes identifies an obviously aberrantly high expression signature specifically in podocytes, indicative of a distinct stressed cellular state (Fig. 3g). In summary, this murine diabetic kidney atlas not only confirms podocyte-specific metabolic dysregulation as a conserved core disease driver but also delivers an unparalleled resource that delineates the cell-type-specific transcriptional landscape, thereby validating a fundamental pathogenic mechanism, identifying novel therapeutic targets, and establishing a robust preclinical model for DN studies.

### Targeting podocyte integrity with ASIV by ferroptosis pathway modulation

**Fig. 4:**
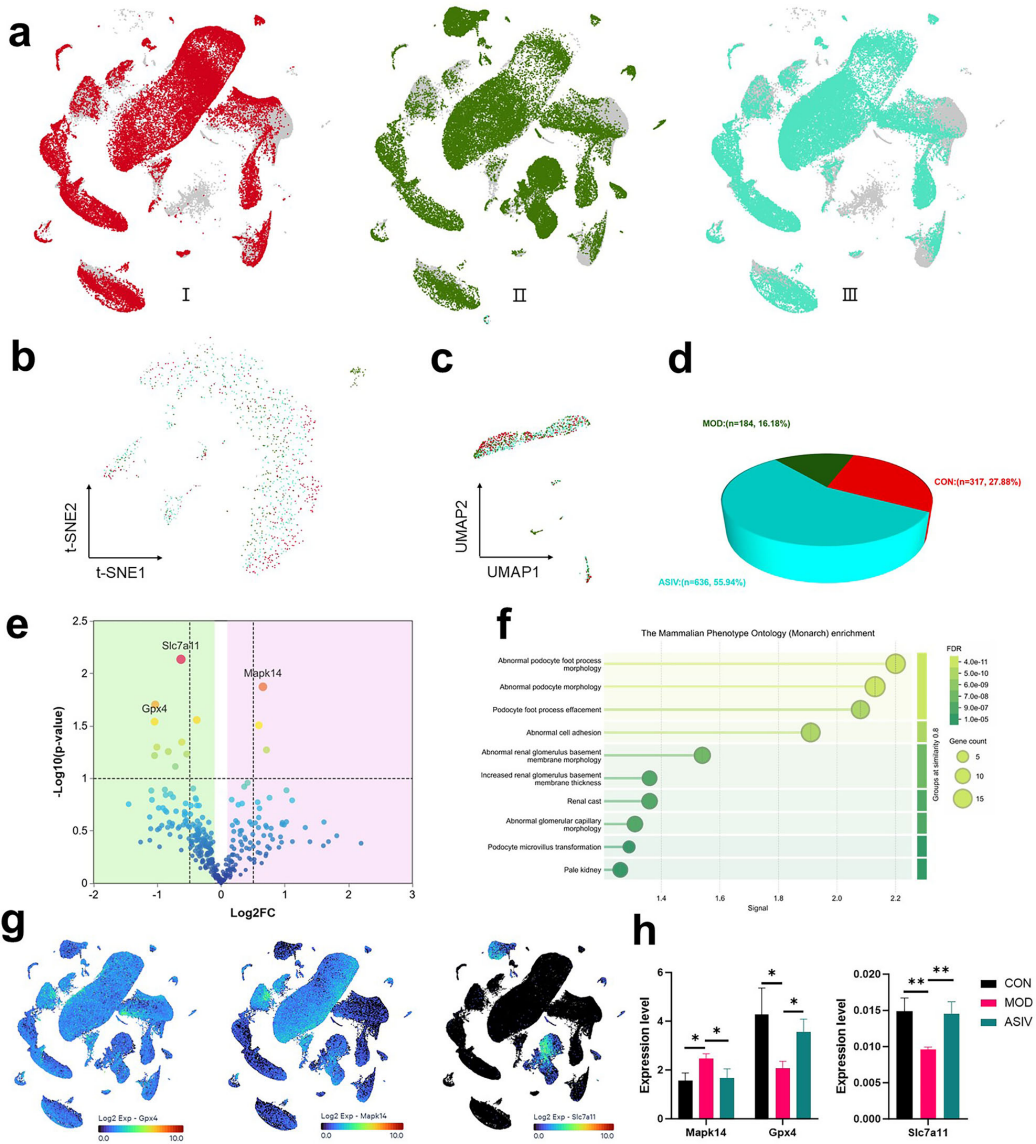
Astragaloside IV attenuates diabetic kidney injury by preserving podocyte homeostasis. **a** Integrated cellular landscape of therapeutic intervention. Uniform Manifold Approximation and Projection (UMAP) visualization of 151,009 high-quality cells from all experimental groups: Group I (non-diabetic db/m mice, *n* = 3), Group II (diabetic db/db mice, *n* = 3), and Group III (diabetic db/db + ASIV-treated, *n* = 3). Cells are colored by experimental condition. High-resolution clustering reveals ASIV-mediated preservation of podocyte integrity. **b** t-SNE and **c** UMAP visualizations of 1137 re-clustered podocytes from all experimental groups, with cells colored by treatment. Both visualizations show that ASIV treatment partially rescues the diabetes-induced transcriptional shift in podocytes. **d** Proportional abundance and quantitative assessment of podocyte across the three experimental groups. ASIV treatment increase the number of podocytes. The db/db mice exhibit significant podocyte loss compared to db/m controls, which is markedly attenuated by ASIV treatment. **e** Volcano plot for the differentially expressed genes in podocyte cells; Significantly regulated genes are highlighted, with key genes annotated. **f** Functional annotation of diabetes mice kidney-induced gene signatures whose diabetes-induced alterations are reversed by ASIV treatment. Bar plot showing the top significantly enriched phenotype ontology terms and the analysis identifies specific morphological and functional deficits in podocytes. **g** UMAP plots showing expression distribution of key genes involved in ferroptosis (GPX4, MAPK14, SLC7A11). **h** Quantitative validation of ASIV-mediated gene regulation. Bar chart comparing normalized expression levels (mean ± SEM; *p < 0.05, **p < 0.01) of ferroptosis-related markers (GPX4, MAPK14, SLC7A11) across experimental groups. ASIV treatment significantly counteracts diabetes-induced alterations in these critical regulators, supporting its role in modulating ferroptosis pathways in diabetic kidney disease. **i** Representative Western Blot bands analyzing key proteins under different treatments. Lanes display levels of phospho-p38 (p-p38), total p38, SLC7A11, GPX4, and LPO, with β-actin or GAPDH as loading controls. Treatments were: Control, Model (high glucose, HG), ASIV (HG + 30 μM ASIV), and C16-PAF (HG + ASIV + 5 μM C16-PAF). Data (mean ± SD; *P < 0.05, **P < 0.01) reveal that HG significantly upregulated p-p38 and LPO while downregulating SLC7A11 and GPX4, AS-IV treatment reversed these changes, and partially blocked by the p38 MAPK activator C16-PAF.

ASIV treatment significantly ameliorated the body weight, hyperglycemia, and HbA1c parameters (*p* < 0.001 and Fig. S1) in experimental mice and confirmed ASIV’s protective effects. To investigate the therapeutic potential of ASIV for preserving podocyte integrity in DN, we employed single-cell transcriptomic analysis of a murine model, focusing on its modulation of the ferroptosis pathway. Initially, UMAP visualization of an integrated cell dataset from non-diabetic, diabetic, and ASIV-treated diabetic mice reveals that therapeutic intervention with ASIV induces a partial reversal of the diabetic cellular landscape, effectively shifting the distribution of renal cells toward a non-diabetic state (Fig. 4a). Subsequently, we demonstrate ASIV-mediated preservation of podocyte integrity through high-resolution clustering, with both t-SNE (Fig. 4b) and UMAP (Fig. 4c) visualizations of 1137 cells clearly showing a partial reversal of the diabetic transcriptional shift, thereby emphasizing the therapeutic potential of ASIV. Furthermore, analysis of podocyte abundance reveals a significant loss in db/db mice versus db/m controls, a key pathological event that is substantially mitigated by ASIV treatment, as quantified by the increase in podocyte numbers, which provides compelling evidence for its role in preserving this critical cell population (Fig. 4d). Moreover, the volcano plot of podocyte-specific DEGs identifies that ASIV induces a broad transcriptomic reprogramming, most notably by significantly regulating key genes such as GPX4, MAPK14, and SLC7A11, which are central regulators of ferroptosis, thereby delineating a potential molecular mechanism (Fig. 4e). Additionally, functional annotation of podocyte genes whose diabetes-induced dysregulation is reversed by ASIV directly links the therapy to the rescue of critical pathological phenotypes, including abnormal podocyte foot process morphology, providing systems-level evidence that ASIV ameliorates the defining structural lesions of diabetic podocytopathy (Fig. 4f). Concurrently, spatial expression of GPX4, MAPK14 and SLC7A11 is mapped at single-cell resolution on UMAP plots, precisely localizing the ferroptosis pathway within distinct states (Fig. 4g). Finally, we quantitatively validate that ASIV counteracts diabetes-induced dysregulation of ferroptosis pathways and demonstrates significant rescue of GPX4, MAPK14, and SLC7A11, establishing a key molecular basis for its therapeutic efficacy (Fig. 4h). This integrated multi-omics investigation definitively establishes ferroptosis inhibition as the principal mechanism of ASIV action by demonstrating a coordinated rescue of critical metabolic imbalances and transcriptional dysregulation, thereby providing a unified molecular framework as a targeted therapy for DN.

### Spatial metabolomics decodes cortex and medulla-specific metabolic dysregulation

**Fig. 5:**
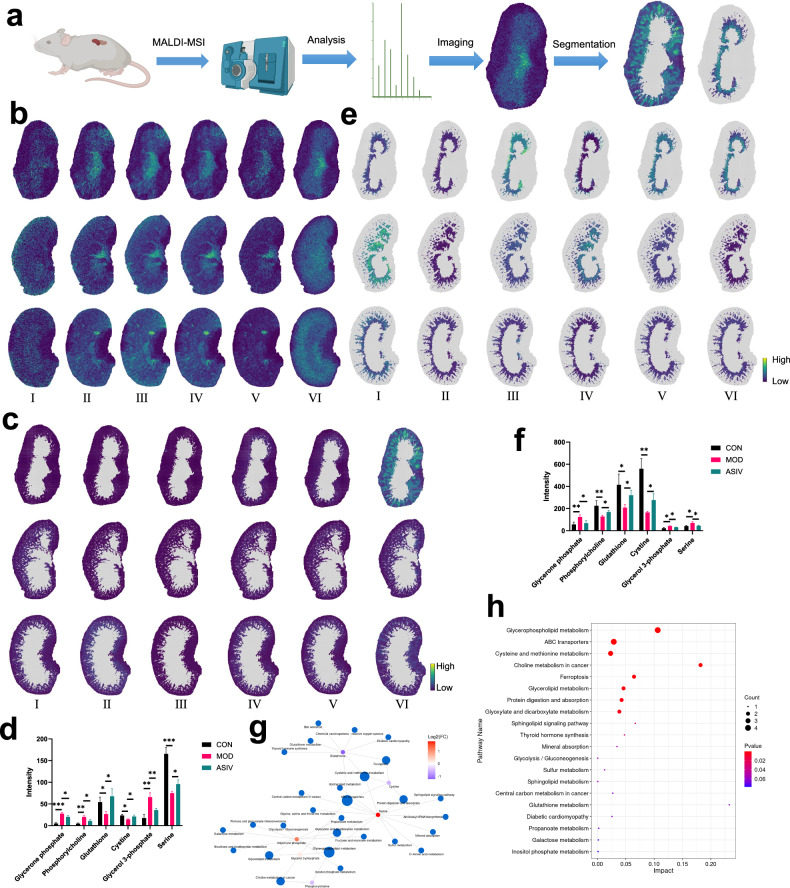
Spatial metabolomics uncovers compartment-specific metabolic dysregulation and its amelioration by ASIV. **a** Experimental workflow for spatial metabolomic profiling. Schematic diagram outlining the key steps: tissue sectioning, matrix application, MS imaging, data preprocessing, segmentation, and annotation. **b** Spatial visualization of diabetes-altered metabolites. Representative ion image showing the anatomical distribution of the significantly dysregulated metabolites (Glycerone phosphate, glycerol 3-phosphate, phosphorylcholine, cystine, glutathione, and serine) in db/db mouse kidney section. **c** Unsupervised segmentation of renal metabolic architecture. Spatial segmentation map generated by applying spatial shrunken centroids clustering to the entire MSI dataset, identifying regions with distinct metabolic profiles. The color-coded regions show high concordance with anatomical cortex structures. **d** Quantification of cortical metabolite dysregulation and therapeutic intervention. Bar plot (mean ± SEM, *n* = 3 biological replicates per group) shows the relative abundance of the metabolite from **c** within a defined cortical region of interest (ROI), which is significantly attenuated by ASIV treatment. (**p < 0.01, ***p < 0.001). **e** Medullary-specific metabolic alteration in diabetes. Spatial distribution image of a distinct metabolite exhibiting intense and specific accumulation in the medullary region of db/db mice, indicating region-specific metabolic stress. **f** Quantitative analysis of medullary metabolite intensity. Bar chart (mean ± SEM) quantifying the normalized intensity of the metabolite from **e** within a medullary ROI across all experimental groups. The marked increase in db/db mice is significantly reduced by ASIV treatment, demonstrating the compound’s efficacy in alleviating medullary metabolic dysfunction. **g** Integrated visualization of the key metabolites and metabolic network across the kidney sections; this visualization highlights coordinated alterations in interconnected metabolic pathways with the key metabolites. **h** Pathway-level interpretation of spatial metabolomic changes. Bar graph showing the results of pathway enrichment analysis (based on KEGG) performed on metabolites differentially abundant in ASIV versus db/db kidneys. The top significantly enriched pathways include glycerophospholipid metabolism, ferroptosis, and glutathione metabolism, directly linking spatial metabolite alterations to known pathogenic mechanisms in diabetic kidney disease.

To decode cortex and medulla-specific metabolic dysregulation in DN, we employed spatial metabolomics to profile the distribution and abundance of key metabolites in murine renal tissues, and accordingly, the experimental workflow for this profiling outlines the key steps from tissue sectioning to data annotation, enabling the visualization of metabolite distribution (Fig. 5a). Subsequently, we reveal the spatial landscape of metabolic alterations through imaging of significantly dysregulated metabolites—such as glycerone phosphate, glycerol 3-phosphate, phosphorylcholine, cystine, glutathione, and serine—in db/db kidney tissues, thereby uncovering region-specific disruptions in critical pathways (Fig. 5b and Table S6). Furthermore, unsupervised segmentation of the renal metabolic architecture identifies distinct regions whose metabolic profiles show high concordance with anatomical structures, most notably revealing a pronounced accumulation of ions in the renal cortex, which underscores a region-specific metabolic reprogramming central to disease progression (Fig. 5c). In support of this, quantification of cortical metabolite dysregulation confirms that diabetes significantly alters key metabolic pathways—elevating Glycerone phosphate (5.59-fold, *p* < 0.001), Glycerol 3-phosphate (3.83-fold, *p* < 0.001), Phosphorylcholine (4.36-fold, *p* < 0.001) while depleting Glutathione (0.49-fold, *p* < 0.05) and Cystine (0.61-fold, *p* < 0.05)—and importantly demonstrates that ASIV treatment significantly attenuates these changes, effectively restoring metabolic homeostasis (Fig. 5d). Concurrently, we identify a medullary-specific metabolic alteration, providing visual evidence of compartmentalized metabolic states (Fig. 5e). Moreover, quantitative analysis of medullary metabolites reveals profound diabetes-induced dysregulation, including increases in glycerone phosphate (2.20-fold, *p* = 0.02) and glycerol 3-phosphate (1.89-fold, *p* = 0.02) alongside decreases in glutathione (0.50-fold, *p* = 0.02), cystine (0.29-fold, *p* < 0.001), and phosphorylcholine (0.57-fold, *p* = 0.02), all of which are significantly attenuated by ASIV treatment (*p* = 0.02–0.04), demonstrating its efficacy in restoring medullary metabolic homeostasis (Fig. 5f). Collectively, visualization of the key metabolite network across the kidney reveals highly organized, spatially defined pathway activities that are systematically disrupted in diabetes, thus demonstrating coordinated alterations in interconnected metabolic processes (Fig. 5g). Consequently, pathway enrichment analysis of spatially resolved metabolites from ASIV-treated kidneys reveals significant alterations in glycerophospholipid metabolism, ferroptosis, and glutathione metabolism, thereby directly linking the therapeutic efficacy of ASIV and providing a metabolic mechanism for its renoprotective effects (Fig. 5h). Ultimately, this study establishes that spatial metabolomics reveals the profound anatomical compartmentalization of metabolic dysregulation, demonstrating that distinct pathogenic alterations specifically affect the renal cortex and medulla, hence providing a mechanistic basis for ASIV’s region-specific therapeutic efficacy.

### Ferroptosis inhibition as the core mechanism of ASIV-mediated podocyte protection

**Fig. 6:**
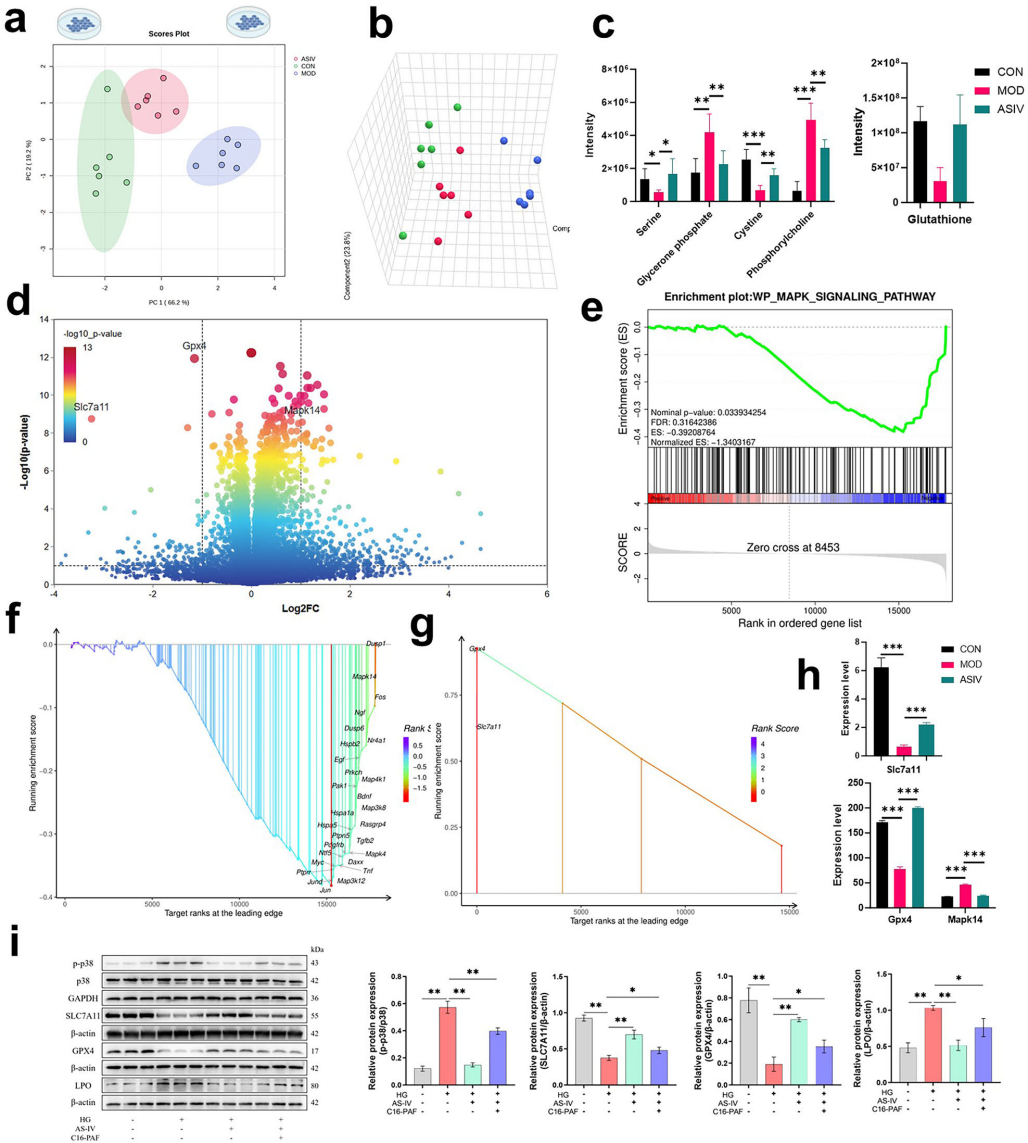
Astragaloside IV attenuates high glucose-induced cellular injury via MAPK signaling and ferroptosis pathways. **a** Principal Component Analysis (PCA) plot, and **b** 3D scatter plot of all MPC-5 podocyte samples across treatment groups: Control (CON, normal glucose), Model (MOD, high glucose), and ASIV Intervention (high glucose+ASIV). Both analyses demonstrate clear separation between groups, indicating that high glucose induces global molecular alterations and that ASIV treatment significantly shifts the metabolomic profiles toward the normal state. **c** Abundance changes of key metabolites involved in high glucose-induced cellular injury. Bar chart (mean ± SEM, *n* = 6 biological replicates; *p < 0.05, **p < 0.01, ***p < 0.001) showing the normalized intensity of critical metabolites. High glucose (MOD) significantly changed the abundance of these metabolites compared to CON, and ASIV treatment significantly restores their levels, indicating a rescue of metabolic function. **d** Transcriptional response to high glucose stress. Volcano plot of differentially expressed genes (DEGs) in MOD versus CON comparison (Log2 fold-change on *x*-axis; -Log10 adjusted *p* value on *y*-axis). Significantly regulated genes are highlighted, with key genes annotated. e Enrichment plot for the ‘MAPK signaling pathway’ gene set (KEGG) in ASIV versus MOD comparison, showing a normalized enrichment score and false discovery rate value. **f** ASIV modulates the MAPK signaling pathway. Gene Set Enrichment Analysis (GSEA) plots showing significant reversal of high glucose-induced alterations in the MAPK signaling pathway by ASIV. **g** Leading edge analysis visualization for the same gene set, highlighting the core genes that contribute most to the enrichment signal. **h** The protective effect of ASIV is mediated through the restoration of GPX4, MAPK14, and SLC7A11 expression under high glucose conditions (mean ± SEM; ***p < 0.001). i Western blot validation of key regulatory genes by ASIV treatment. Bar chart (mean ± SEM; *p < 0.05, **p < 0.01) comparing the normalized expression of marker genes GPX4, MAPK14, and SLC7A11 across CON, MOD, and ASIV groups. High glucose significantly alters the expression of these ferroptosis and MAPK pathway regulators, and ASIV treatment significantly counteracts these changes, confirming the mechanistic role of ASIV against podocyte injury.

To elucidate the core mechanism of ASIV-mediated podocyte protection, we performed integrated metabolomic and transcriptomic profiling, which identified ferroptosis inhibition as the central therapeutic action. PCA and 3D scatter plots revealed distinct clustering of podocyte samples (Fig. 6a, b) under control (CON), high glucose (MOD), and ASIV intervention conditions, thereby demonstrating that high glucose induces profound global molecular alterations that are significantly reversed by ASIV treatment. Subsequently, Targeted metabolomic analysis reveals that high glucose exposure induces significant dysregulation of key metabolites in podocytes - depleting glutathione (0.26-fold, *p* < 0.001), serine (0.40-fold, *p* < 0.05), and cystine (0.28-fold, *p* < 0.001) while elevating glycerone phosphate (2.38-fold, *p* < 0.01) and phosphorylcholine (7.54-fold, *p* < 0.001)—alterations that are significantly rescued by ASIV treatment (*p* < 0.05–0.01), thereby demonstrating its efficacy in restoring metabolic homeostasis (Fig. 6c). Concurrently, transcriptional profiling under high glucose stress revealed widespread differential gene expression, with a volcano plot specifically annotating key dysregulated genes *GPX4*, *MAPK14*, and *SLC7A11*, thus implicating ferroptosis as a central mechanism in glucose-induced injury (Fig. 6d). Furthermore, Gene Set Enrichment Analysis demonstrated that ASIV significantly modulates the MAPK signaling pathway, with an enrichment plot showing a marked reversal of the diabetic signature (Fig. 6e), while the lead edge analysis pinpointed the core genes driving this protective transcriptional reprogramming (Fig. 6f). Critically, we identified the reversal of the ferroptosis process as the primary functional outcome of ASIV treatment, based on its significant enrichment among rescued genes (Fig. 6g). Finally, we found that ASIV’s protective effect is mediated through the significant reversal of high glucose-induced alterations in *GPX4*, *MAPK14*, and *SLC7A11* (Fig. 6h). Western blot analysis validated that HG exposure significantly increased phosphorylated p38 MAPK14 (p-p38) and lipid peroxidation (LPO) levels, while downregulating key ferroptosis-related proteins SLC7A11 and GPX4, indicating HG-induced ferroptosis activation (Fig. 6i). Treatment with AS-IV effectively reversed these changes, reducing p-p38 and LPO expression and restoring SLC7A11 and GPX4 levels. Furthermore, the addition of the p38 MAPK activator C16-PAF partially abolished the protective effects of AS-IV, confirming that AS-IV alleviates ferroptosis by inhibiting the p38 MAPK pathway. These findings suggest that AS-IV protects podocytes from HG-induced damage by modulating the p38 MAPK/SLC7A11/GPX4 axis.

### Clinical metabolomic profiling reveals urinary metabolite biomarkers

**Fig. 7:**
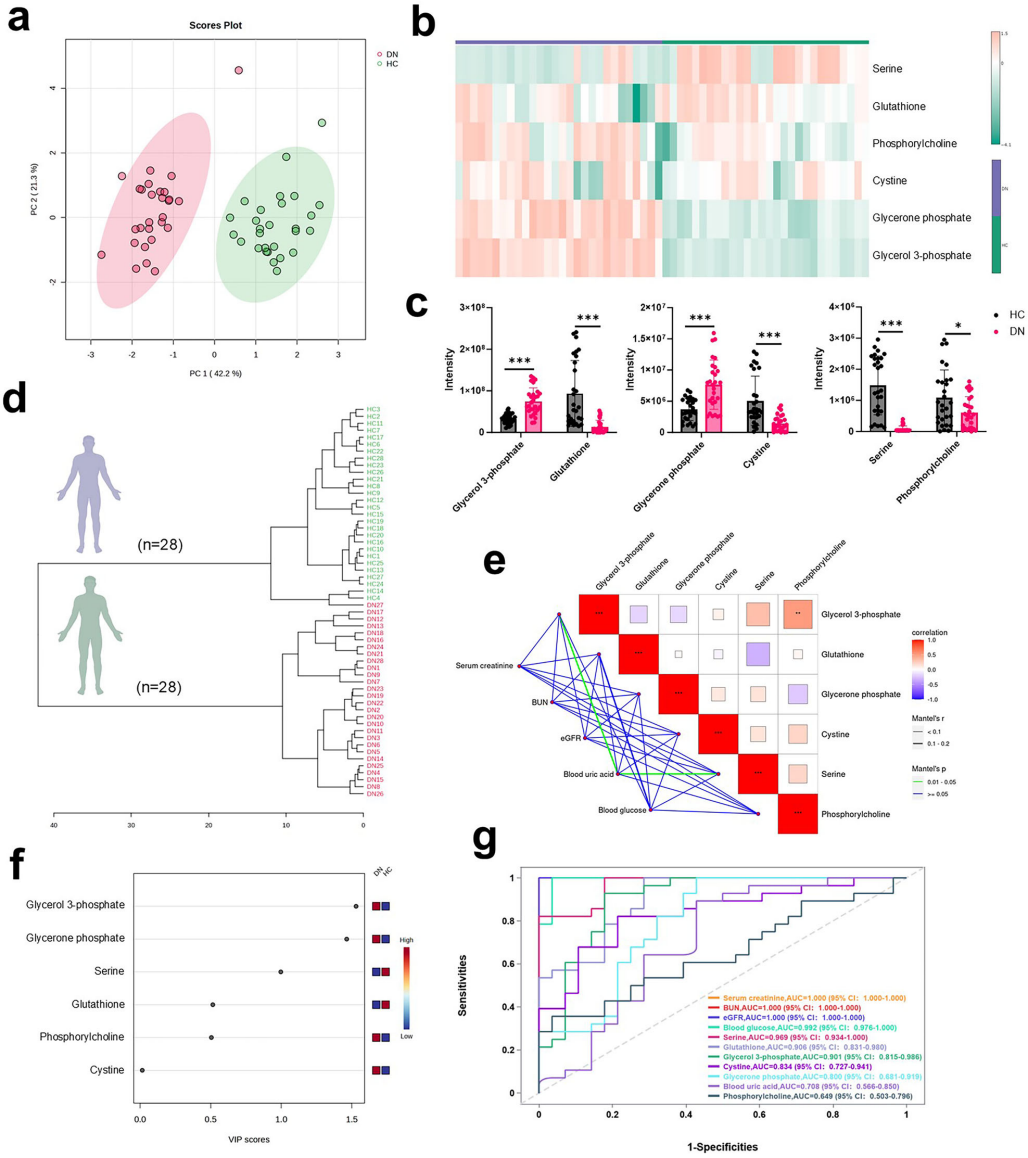
Clinical metabolomic profiling reveals urinary relevant biomarkers. **a** Global metabolic separation between patient groups. Principal Component Analysis (PCA) score plot of urinary metabolomic profiles from 28 healthy controls (HC) and 28 DN patients. Clear separation along principal component 1 (PC1, 42.2%) and PC2 (21.3%) indicates distinct global metabolic phenotypes, reflecting the systemic metabolic disruption in DN. **b** Heatmap displaying the normalized abundance (Z-score) of significantly altered metabolites (rows) across all individual samples (columns). Metabolites and samples are hierarchically clustered, revealing coherent metabolic patterns that robustly distinguish DN patients from healthy controls. **c** Quantitative overview of metabolic dysregulation. Bar chart showing the number of significantly upregulated and downregulated metabolites in the DN group compared to HC. **d** Horizontal clustering analysis performed on the significantly dysregulated metabolites. Each row represents a metabolite, and columns represent sample groups (HC, DN). The color intensity reflects relative abundance, providing a visual fingerprint of the DN-specific metabolic state. **e** Integration of metabolomic data with clinical parameters. Correlation analysis between the significant differential metabolites and key clinical indices of renal function (Serum creatinine, BUN, eGFR, Blood uric acid, Blood glucose). The color scale represents the Spearman correlation coefficient (*r*), with asterisks denoting statistical significance (*p < 0.05, **p < 0.01, ***p < 0.001). Strong correlations highlight the clinical relevance of the discovered metabolites. **f** Variable Importance in Projection (VIP) plot from the orthogonal Partial Least Squares-Discriminant Analysis (PLS-DA) model comparing DN and HC groups. Metabolites are ranked by their VIP score, with those exceeding the threshold of 1.0 considered highly influential in separating the two cohorts. **g** Diagnostic potential of candidate metabolite biomarkers. ROC curves evaluating the ability of individual top-ranked metabolites to discriminate DN patients from healthy controls. The AUC for each metabolite is indicated and several metabolites show high diagnostic potential (AUC > 0.90), suggesting their utility as non-invasive biomarkers for DN.

To identify non-invasive urinary metabolite biomarkers for diagnosing DN, we conducted a clinical metabolomic profiling study comparing patients with healthy controls. Initially, PCA of urinary metabolomes revealed a clear separation between DN patients (*n* = 28) and healthy controls (*n* = 28) along principal components 1 (42.2%) and 2 (21.3%), indicating distinct global metabolic phenotypes reflective of systemic metabolic disruption in DN (Fig. 7a). Subsequently, hierarchical clustering of significantly altered metabolites across all individual samples showed coherent metabolic patterns that robustly distinguish DN patients from controls, providing a visual and analytical foundation for identifying metabolite signatures (Fig. 7b). Furthermore, a bar chart delineating the scale of metabolic alterations quantified the total number of significantly upregulated and downregulated metabolites (Fig. 7c). Similarly, horizontal clustering analysis of these dysregulated metabolites generated a distinct visual fingerprint of the DN metabolic state, clearly differentiating patients from healthy controls (Fig. 7d). To establish clinical relevance, correlation analysis between dysregulated metabolites and clinical parameters identifies significant associations, including between blood uric acid and glycerol 3-phosphate/serine, directly linking the discovered metabolic alterations to impaired renal function in DN patients (Fig. 7e and Table S7). Moreover, the variable importance in projection (VIP) plot from the PLS-DA model identified glycerol 3-phosphate and glycerone phosphate as the most important metabolites (VIP > 1.0) for group separation, pinpointing them as key components of the disease’s metabolic profile (Fig. 7f). Most importantly, evaluation of diagnostic potential reveals that specific urinary metabolites exhibit exceptional ability to discriminate DN patients, with serine (AUC = 0.969), glutathione (AUC = 0.906), and glycerol 3-phosphate (AUC = 0.901) showing AUC values surpassing 0.90, rivaling even clinical standards like blood glucose (AUC = 0.992) and significantly outperforming blood uric acid (AUC = 0.708), thereby positioning these metabolites as powerful non-invasive biomarkers for disease detection (Fig. 7g). In conclusion, this study successfully identifies and validates a panel of urinary metabolites as highly sensitive and specific non-invasive biomarkers for DN, providing a powerful tool for early detection and risk stratification which could significantly improve clinical management.

## Discussion

This study delivers a comprehensive, multi-dimensional analysis of DN pathogenesis, spanning from single-cell resolution in human and murine kidneys to spatial metabolomics and clinical validation. By integrating snRNA-seq of patient biopsies with a high-resolution murine model and interventional studies on ASIV, we decode the cellular ecosystem of DN. Our central finding is the identification of podocyte ferroptosis, driven by interconnected disruptions in glycerophospholipid metabolism, as a paramount mechanism of glomerular injury. This pathway-specific, conserved across species and ameliorated by ASIV, not only redefines our understanding of podocyte loss but also unveils a suite of highly sensitive urinary metabolite biomarkers, charting a course from fundamental mechanism discovery to clinical diagnostic and therapeutic advancement.

Our human DN cellular atlas provides the first truly comprehensive view of the diseased renal landscape, moving beyond cataloging cell types to reveal the podocyte as the transcriptional core of DN. The superior diagnostic power of the podocyte-specific signature, outperforming all other renal cell types, underscores its key role in disease pathogenesis. This finding challenges a purely tubular-centric view of DN progression and aligns with emerging evidence that early podocyte injury is a critical determinant of diabetic glomerulopathy [19, 20]. The pathway enrichment analysis revealing ferroptosis, glycerophospholipid metabolism, and glutamatergic synapse as top dysregulated pathways in podocytes is particularly insightful. While oxidative stress is a recognized feature of DN, our data precisely implicate ferroptosis, an iron-dependent, non-apoptotic cell death pathway characterized by glutathione depletion and lipid peroxidation [11]. The significant dysregulation of key ferroptosis regulators (*SLC7A11*, *GPX4*) and associated metabolic pathways provides a mechanistic link between the DN and podocyte demise. Furthermore, the dysregulation of glycerophospholipid metabolism directly potentiates lipid peroxidation and ferroptosis. Glycerophospholipids constitute the primary lipid bilayer and are enriched with polyunsaturated fatty acids (PUFAs). Their metabolic perturbation alters membrane composition, increasing the PUFAs level. These PUFAs are prime substrates for lipid peroxidation initiated by reactive oxygen species, thereby directly fueling the lethal lipid peroxide accumulation that defines ferroptosis execution. Recent work by Wang et al. showed at ferroptosis in renal tubules in diabetes [21], but our single-cell data unequivocally reveal the podocyte as the primary glomerular target, a notion further strengthened by the pronounced heterogeneity within this population, suggesting distinct states of susceptibility to this form of death. The connection to glutamatergic synapse pathways, while unexpected, may reflect aberrant signaling in the podocyte’s intricate cellular architecture, potentially related to metabolic homeostasis, requiring further study [22].

The conservation of this podocyte-specific metabolic dysregulation in our murine diabetic kidney atlas is a critical validation step, bridging human pathology with a tractable experimental model. The recapitulation of glycerophospholipid metabolism pathway alterations in mouse podocytes confirms these as core, evolutionarily conserved drivers of disease. Glycerophospholipids are fundamental components of cellular membranes, and their dysregulation directly threatens podocyte structural integrity, leading to foot process effacement [23]. Our spatial metabolomics data powerfully extend this concept by demonstrating that these metabolic disruptions are not uniform but are anatomically compartmentalized. The profound accumulation of glycerone phosphate and glycerol 3-phosphate in the cortex, where glomeruli reside, directly mirrors the transcriptomic findings and spatially localizes the metabolic insult to the podocyte’s environment. Conversely, the medulla exhibits a distinct metabolic profile, highlighting region-specific vulnerabilities. The depletion of glutathione and its precursor, cystine, in both cortex and medulla, but particularly in the cortex, creates a perfect storm for ferroptosis, as the cystine/glutamate antiporter is essential for glutathione synthesis and antioxidant defense [24]. This integrated transcriptomic-metabolomic framework resolves a long-standing paradox in DN: how generalized metabolic insults manifest as highly specific cellular injury.

The most significant therapeutic implication of our work is the demonstration that ASIV, a natural compound with known renoprotective properties, exerts its effect primarily by inhibiting podocyte ferroptosis. Our data provide a complete chain of evidence: from the single-cell transcriptomic reversal of the diabetic landscape in mice, to the specific preservation of podocyte numbers, and down to the precise reprogramming of the core ferroptosis pathway. The functional annotation showing ASIV’s reversal of terms like “Abnormal podocyte foot process morphology” provides systems-level validation that this molecular rescue translates to structural preservation. This positions ASIV not as a general antioxidant but as a targeted ferroptosis inhibitor. This is a novel mechanism of action for this compound and aligns with the growing interest in targeting ferroptosis in other degenerative diseases [12, 14]. The role of MAPK14 (p38α) is particularly intriguing, as it integrates stress signals with cell fate decisions and has been implicated in other forms of renal injury [25]; our data suggest it may be a key node linking diabetic stress to the ferroptosis in podocytes. The efficacy of ASIV in restoring spatial metabolic homeostasis in both cortex and medulla further underscores its potential as a multi-compartment therapeutic agent.

Finally, the translation of our mechanistic findings into a clinically diagnostic tool represents a major step toward personalized medicine for DN. The identification of urinary serine, glutathione, and glycerol 3-phosphate as biomarkers with exceptional diagnostic power is groundbreaking. These metabolites are not arbitrary markers; they are direct reflections of the pathogenic pathways we identified-glycerol 3-phosphate in glycerophospholipid metabolism and serine/glutathione in the ferroptosis axis. Their performance is comparable with that of blood glucose, the hallmark of diabetes, and significantly outperforms standard renal function markers like blood uric acid. This suggests that these metabolites capture the specific renal tissue pathology of DN rather than just systemic dysglycemia. The correlation between these metabolites and clinical parameters like blood uric acid strengthens their biological plausibility and clinical relevance. Recent studies have begun to explore metabolomics in DN [26, 27], but our study is unique in linking specific urinary metabolites back to a defined cell-type-specific pathway disruption via an integrated multi-omics approach. This offers a non-invasive window into podocyte health, potentially enabling early diagnosis before significant albuminuria or functional decline, and could serve as a pharmacodynamic biomarker for assessing the efficacy of podocyte-protective therapies like ASIV.

In conclusion, our study moves beyond descriptive omics to establish a causal pathway from diabetic insult to podocyte loss via ferroptosis. We redefine DN by highlighting the podocyte’s metabolic dysregulation, provide a robust preclinical rationale for targeting ferroptosis with ASIV, and deliver a practical diagnostic biomarker panel derived directly from the core disease mechanism. This work not only fundamentally advances our understanding of DN pathobiology but also provides a comprehensive framework for developing mechanistically grounded diagnostics and therapeutics, addressing a critical need in the management of this devastating disease.

This study has several limitations. Firstly, while integrative, the primary human snRNA-seq analysis was conducted on a relatively small cohort, which may limit the generalizability of the identified podocyte-centric signatures. Although validated in mice and clinical metabolites, the causal link between the MAPK14/SLC7A11/GPX4 axis dysregulation and podocyte ferroptosis, while strongly supported by in vitro and intervention data, would benefit from further genetic loss-of-function experiments in vivo. Secondly, the protective mechanism of ASIV, though comprehensively mapped, was primarily explored in mice model and cell line; its long-term efficacy in humans require further investigation. Finally, the promising urinary biomarkers, while exhibiting high diagnostic accuracy, were identified in a single, modest-sized cohort and need validation in larger, independent, and multi-center populations to confirm their clinical utility.

## Conclusion

Our study establishes a new pathogenic axis in DN, centering on podocyte ferroptosis driven by dysregulated glycerophospholipid metabolism. Through integrated multi-omics across human and murine systems, we have identified this pathway as a conserved core mechanism of glomerular injury. We further demonstrate that ASIV exerts potent renoprotection by specifically inhibiting ferroptosis, reversing transcriptional and metabolic aberrations in podocytes, and restoring spatial metabolic homeostasis. Crucially, we translate these mechanistic insights into the clinical field by validating a panel of urinary metabolites - serine, glutathione, and glycerol 3-phosphate - as highly sensitive and specific non-invasive biomarkers for DN. This work redefines DN pathophysiology by highlighting podocyte metabolic disorders, provides a compelling therapeutic strategy via ferroptosis inhibition, and delivers a practical diagnostic tool, thereby bridging fundamental discovery to the clinical advancement for DN disease.

## Methods

### Human subjects and study approval

This study was conducted in compliance with the Declaration of Helsinki and approved by the Institutional Review Board of the First Affiliated Hospital of Hainan Medical University (Ethical Approval No. ChiCTR2400087438). We have enrolled 31 patients with DN and 28 age- and sex-matched healthy controls (HC). Urine samples from all participants were subjected to targeted metabolomic profiling. Additionally, formalin-fixed paraffin-embedded (FFPE) renal tissues were collected from DN patients for single-nucleus RNA sequencing (snRNA-seq) to enable high-resolution transcriptomic analysis of human kidney.

The DN and HC cohorts were well-matched in age and sex distribution (Supplementary Table S1). DN group displayed the expected clinical hallmarks of severe renal dysfunction, including significantly elevated serum creatinine, blood urea nitrogen (BUN), uric acid, and fasting blood glucose levels (all *p* < 0.01), alongside a profoundly reduced estimated glomerular filtration rate (eGFR) (*p* < 0.0001) compared to controls. This clinical profile confirms the enrollment of a well-defined DN patient group with significant renal impairment, providing a robust foundation for subsequent molecular investigations.

### scRNA-seq transcriptomic profiling analysis

Human kidney tissue specimens were freshly collected, fixed in 4% formalin, and embedded in paraffin (FFPE) using standard pathological procedures. Tissue blocks were sectioned at 5 µm thickness for scRNA-seq transcriptomic analysis. All subsequent steps were carried out in accordance with the 10x Genomics Visium HD FFPE Tissue Preparation Handbook (CG000684). Following image acquisition, gene barcoding was performed using the Gene Expression Reagent Kit (10× Genomics, PN-1000675) as described in the user manual (CG000685). The barcoded cDNA libraries were prepared, quantified, and sequenced on an Illumina platform under a paired-end 150 bp configuration.

### scRNA-seq data processing and alignment

We processed raw sequencing reads using the 10× Genomics Ranger pipeline (v3.0.1) for alignment to the GRCh38 human reference genome. The pipeline handled demultiplexing, read alignment, UMI counting, and generation of a gene-spot count matrix. Tissue-covered spots were identified automatically by applying the integrated tissue detection algorithm in Space Ranger. The filtered gene expression matrix was imported into R (v4.0.3) and analyzed using the Seurat package (v5.1.0).

### scRNA-seq data normalization and feature selection

Quality control metrics—including UMIs per spot and genes detected—were evaluated to exclude low-quality or outlier spots. Normalization was performed using the “LogNormalize” method in Seurat, which scales expression values by the total UMI count per spot, applies a factor of 10,000, and log-transforms the result. We next identified the top 3000 highly variable genes using the FindVariableFeatures function with variance-stabilizing transformation. These genes were used in downstream dimensional reduction and clustering steps.

### Dimensionality reduction and unsupervised clustering

Principal component analysis (PCA) and partial least squares-discriminant analysis (PLS-DA) model was applied to the scaled HVG expression matrix to reduce dimensionality. Cell clusters were identified using graph-based clustering via FindClusters function, with resolution tuned to optimize cluster distinction. Cluster relationships were visualized in two dimensions using Uniform Manifold Approximation and Projection (UMAP). Putative cell-type identities were assigned by identifying cluster-specific marker genes with FindAllMarkers and comparing them to established renal cell-type signatures. The diagnostic model was constructed based on cell-type-specific gene expression signatures derived from single-nucleus RNA sequencing data of renal biopsies. For each major cell type, DEGs were identified and used as features. Model performance was evaluated via receiver operating characteristic (ROC) analysis with the area under the curve (AUC).

### Differential expression and pathway analysis

DEGs between conditions or regions were detected using FindMarkers. Significance thresholds were set at an adjusted *p*-value < 0.05 and |log₂FC| > 1.

Functional enrichment analysis of DEGs was conducted using the clusterProfiler R package, which tested for over-representation of Gene Ontology (GO) terms and Kyoto Encyclopedia of Genes and Genomes (KEGG) pathways based on a hypergeometric distribution model.

### Metabolomic profiling of urine from DN patients

Following thawing at 4 °C, urine aliquots were vortex-mixed and subjected to metabolite extraction using 80% methanol containing 2-chloro-L-phenylalanine (4 ppm) as an internal standard. After centrifugation (12,000 rpm, 10 min, 4 °C), the supernatant was passed through a 0.22 μm PTFE membrane filter. Metabolite separation was performed on a Vanquish UHPLC platform equipped with an HSS T3 column (2.1 × 100 mm, 1.8 μm), using a gradient elution with acetonitrile containing 0.1% formic acid in positive ion mode (ESI+) or acetonitrile with 10 mM ammonium formate in negative ion mode (ESI−), ranging from 8% to 98% organic phase. Mass spectrometry analysis was conducted on an Orbitrap Exploris 120 instrument operating in full scan/data-dependent MS² acquisition mode (MS1 resolution: 60,000; MS2 resolution: 15,000; scan range: m/z 100–1000). Raw data were converted to mzXML format using ProteoWizard and processed via XCMS for peak picking, alignment, and quality control (relative standard deviation, RSD < 30%). Metabolite identification was performed by matching against the HMDB, KEGG, and a custom mass spectral database.

### Development and validation of diagnostic signatures

A diagnostic model was constructed based on urinary targeted metabolomics (LC-MS) data, which quantified metabolic intermediates in 28 DN patients and 28 matched controls. The model’s discriminatory power was evaluated by receiver operating characteristic (ROC) analysis implemented with the R package pROC, using the area under the curve (AUC) to measure predictive accuracy. Final validation was conducted via the CNSknowall platform, which supports integrated biomedical data visualization and statistical modeling.

### Animal studies

All experimental procedures were approved by the Animal Ethics Committee of Hainan Medical University (Protocol HYLL-2023-457) and conducted in compliance with institutional guidelines. Male C57BL/KsJ db/m (non-diabetic) and db/db (diabetic) mice (7 weeks old) were obtained from Jiangsu Jicui Yaokang Biotechnology Co., Ltd (China; license SCXK-2023-0009). The mice were randomly assigned to three experimental groups (*n* = 6 per group): a control (CON) group (db/m mice receiving saline), a diabetic model (MOD) group (db/db mice receiving saline), and an intervention group (db/db mice administered Astragaloside IV (ASIV) at 1 g/kg/day) [28, 29]. All treatments were administered once daily by oral gavage for a duration of 4 weeks. Following the treatment period, mice were anesthetized, and kidney tissues were collected, embedded in carboxymethyl cellulose (CMC), snap-frozen in liquid nitrogen, and stored at −80 °C for subsequent analysis.

### Kidney tissue embedding, sectioning, and quality control for MALDI-MSI

Mouse kidney tissues were embedded in a 2% carboxymethyl cellulose (CMC) matrix under cryogenic conditions using an isopentane-dry ice bath to preserve metabolic integrity. Consecutive tissue sections (10 µm thickness) were collected from each embedded block. To assess technical reproducibility, sections from the same tissue block were collected on separate days and processed independently as technical replicates. All sections were mounted on pre-cooled indium tin oxide (ITO)-coated slides and stored at –80 °C until analysis.

### Matrix coating and MALDI-mass spectrometry imaging

Prior to matrix application, 2-chloro-L-phenylalanine (4 ppm) was homogeneously sprayed as an internal standard for signal normalization and quality control across replicates. The 9-aminoacridine matrix (10 mg/mL in 70% ethanol) was applied using an HTX TM sprayer under standardized conditions. MALDI-MSI was performed on a tims-TOF flex instrument in negative ion mode. Each imaging run included a pooled quality control (QC) section composed of tissue segments from all experimental groups to monitor instrument stability. Pixel-level intensity variation was assessed between technical replicates, with median coefficient of variation (CV) maintained below 20% for key metabolites.

### Data processing, metabolite identification, and reprodubility validation

Raw data were processed in SCiLS™ Lab 2024, including spectral alignment and normalization using the internal standard. Metabolites were annotated via the METASPACE platform querying HMDB, LipidMaps, CoreMetabolome, and KEGG, with a false discovery rate (FDR) < 10%. Identification confidence was increased by matching measured m/z values within 5 ppm mass error and validating with isotopic pattern consistency. Spatial segmentation and differential analysis were performed, and reproducibility was further confirmed by high Pearson correlation between technical replicates for region-specific ion intensities.

### Single-cell RNA sequencing analysis of mice kidney

Single-cell RNA sequencing data were processed using the Cell Ranger pipeline (v9.0.0; 10× Genomics) for alignment to the GRCm39 mouse genome and quantification of unique molecular identifiers (UMIs). Subsequent analysis was performed with the Seurat package (v4.0.0) in R. Low-quality cells and potential multiplets were filtered out based on the following thresholds: genes detected per cell <200, total UMI counts <0.7, mitochondrial gene content >10%, and hemoglobin gene content >5%. Putative doublets were further identified and removed using the DoubletFinder algorithm (v2.0.3). Gene expression levels were normalized using a global-scaling approach, which scales counts per cell to a factor of 10,000 followed by natural-log transformation.

### Dimensionality reduction, clustering, and functional enrichment

The top 2000 highly variable genes were selected for dimensionality reduction via principal component analysis (PCA). Cell clusters were identified using a graph-based clustering algorithm, and the resulting populations were visualized in two dimensions using Uniform Manifold Approximation and Projection (UMAP). Cluster-specific marker genes and DEGs between conditions were identified using presto-based methods, with significance thresholds set at an adjusted *p*-value < 0.05 and an absolute log2-fold change >0.58. Significant DEGs were subsequently subjected to GO and KEGG pathway enrichment analyses in R (v4.0.3) using a hypergeometric test.

### Cell culture and treatment

Mouse podocyte clone MPC-5 cells were cultured in Dulbecco’s Modified Eagle Medium (DMEM) supplemented with 10% fetal bovine serum (FBS) and 1% penicillin-streptomycin, under standard conditions (37 °C, 5% CO_2_). At 70–80% confluency, cells were subjected to 24-h serum-free treatments across four experimental groups: normal glucose (CON, control), high glucose (MOD, 60 mM), and high glucose plus 30 μM ASIV [30, 31, 32] Following treatment, cells were washed with phosphate-buffered saline (PBS), lysed with TRIzol reagent, and snap-frozen in liquid nitrogen for subsequent RNA sequencing. Cell viability and morphological changes were regularly assessed using an inverted microscope. Each condition included six biological replicates to ensure statistical reliability. Transcriptomic profiling was employed to investigate high glucose-induced injury mechanisms and the protective effects of the tested compounds.

### Cellular metabolomic analysis in MPC-5 cells

MPC-5 cell metabolites were extracted with acetonitrile:methanol:water (2:2:1) containing 2-chloro-L-phenylalanine (4 ppm), homogenized (bead-beating, 60 Hz, 2 min), and centrifuged. The extract was dried, reconstituted, filtered (0.22 μm PTFE), and analyzed by UPLC-Orbitrap MS (HSS T3 column; gradient elution with 0.1% formic acid or 5 mM ammonium formate; m/z 100–1000). Data were processed (XCMS, RSD < 30%), and metabolites were annotated using HMDB, KEGG, and LipidMaps for subsequent differential analysis.

### Western blot analysis

Western blot experiment was conducted to investigate the effect of AS-IV on high glucose (HG)-induced podocyte injury via the ferroptosis pathway. Immortalized mouse podocytes (MPC-5) were cultured in DMEM supplemented with 10% FBS and 1% penicillin-streptomycin. Cells were divided into four groups: Control, Model (60 mM HG), ASIV (60 mM HG + 30 μM ASIV), and C16-PAF (60 mM HG + 30 μM ASIV + 5 μM C16-PAF). After treatment, total protein was extracted using RIPA lysis buffer with protease and phosphatase inhibitors. Protein concentration was determined using a BCA assay. Samples (20 μg per lane) were separated by SDS-PAGE and transferred to PVDF membranes. After blocking, membranes were incubated overnight at 4 °C with primary antibodies against p-p38(14064-1-AP, Proteintech Group, China), SLC7A11(T57046, Abmart, China), GPX4(67763-1-Ig, Proteintech Group, China), LPO(10376-1-AP, Proteintech Group, China), and β-actin(B009, Biodragon, China)/GAPDH(26520-1-AP, Proteintech Group, China), followed by HRP-conjugated secondary antibodies. Protein bands were visualized using an ECL detection system and quantified with ImageJ software. Relative protein expression was normalized to β-actin or GAPDH.

### mRNA sequencing and bioinformatic analysis

Total RNA was isolated from MPC-5 podocytes using TRIzol reagent, and its integrity was verified using a NanoDrop 2100 spectrophotometer and a Bioanalyzer. Sequencing libraries were constructed with the VAHTS Universal V6 Kit and sequenced on an Illumina NovaSeq 6000 platform to generate 150-bp paired-end reads. Raw sequencing data were subjected to quality control, aligned to the GRCh38 reference genome using HISAT2, and gene expression levels were quantified as FPKM and raw read counts with HTSeq. Differential gene expression analysis was performed to identify statistically significant transcripts.

Functional interpretation of the results included GO and KEGG pathway enrichment analyses based on the hypergeometric distribution to identify overrepresented biological themes. Gene Set Enrichment Analysis (GSEA) was further applied to assess coordinated changes in predefined gene sets. Finally, protein-protein interaction networks were inferred from the DEGs using the STRING database.

### Statistical analysis

All statistical analyses were performed using R (version 4.1.2) and GraphPad Prism (version 10). Data are expressed as mean ± standard error of the mean (SEM). Differences between groups were assessed using two-tailed Student’s *t* tests in GraphPad Prism (v10.0), and *p*-values were adjusted for multiple comparisons using the Benjamini–Hochberg false discovery rate (FDR) method. A corrected *p*-value of less than 0.05 was considered statistically significant.

## Supplementary information

Supplementary Information

Original Data Files

## Data Availability

The data that support the findings are included within the supplementary material.
