# Supplementary material for: MAPK14/SLC7A11/GPX4 axis dysregulation drives podocyte ferroptosis via mediating glycerophospholipid metabolism
Source: Cell Death Discov. 2026 Mar 11;12:147. doi: 10.1038/s41420-026-02990-7 (PMC13039714; doi:10.1038/s41420-026-02990-7)
Supplement: Supplementary file 1 — Supplementary Information [file 41420_2026_2990_MOESM1_ESM.docx]

**Table S1 The clinical features of all the participants.**

| **ID** | **DN** | **HC** | **P-value** |
| --- | --- | --- | --- |
| Number | 31 | 28 | - |
| Female/Male | 8/23 | 9/19 | - |
| Age | 58.8710±9.6047 | 57.9643±9.6896 | 0.7198 |
| BMI (kg/m2) | 23.5305±2.7643 | 25.1058±4.8950 | 0.1288 |
| Serum creatinine(μmol/l) | 476.1968±246.6314 | 70.6464±13.7990 | 0.0000 |
| BUN(mmol/l) | 20.4113±8.3824 | 4.5925±1.3403 | 0.0000 |
| eGFR(mL/min/1.73m2) | 16.0000±13.8778 | 100.0357±13.5250 | 0.0000 |
| Blood uric acid(μmol/l) | 441.0714±109.8194 | 365.7500±65.0491 | 0.0029 |
| Fasting blood glucose(mmol/l) | 9.0713±2.5764 | 5.0279±0.5059 | 0.0000 |

Note: BUN: blood urea nitrogen; eGFR: estimated glomerular filtration rate.

**Table S2 Functional pathway enrichment analysis of thr significantly enriched KEGG pathways for each major cell type in DN.**

| **Type** | **Descrption** | **Value** |
| --- | --- | --- |
| Erythroblast | Calcium signaling pathway | 0.6417 |
| Erythroblast | cGMP-PKG signaling pathway | 0.6627 |
| Erythroblast | MAPK signaling pathway | 0.5800 |
| Erythroblast | Ras signaling pathway | 0.6050 |
| Erythroblast | Oxytocin signaling pathway | 0.6258 |
| Loop of Henle cell | cAMP signaling pathway | 0.5752 |
| Loop of Henle cell | Apelin signaling pathway | 0.6286 |
| Loop of Henle cell | Cytoskeleton in muscle cells | 0.5708 |
| Loop of Henle cell | Phospholipase D signaling pathway | 0.6174 |
| Loop of Henle cell | Notch signaling pathway | 0.7419 |
| Classical B cell | Relaxin signaling pathway | 0.5692 |
| Classical B cell | Chemokine signaling pathway | 0.5337 |
| Classical B cell | Neurotrophin signaling pathway | 0.5750 |
| Classical B cell | Adipocytokine signaling pathway | 0.5857 |
| Classical B cell | Endocytosis | 0.4841 |
| Cortical stromal cell | Sphingolipid signaling pathway | 0.5246 |
| Cortical stromal cell | Thyroid hormone signaling pathway | 0.5246 |
| Cortical stromal cell | Cortisol synthesis and secretion | 0.5692 |
| Cortical stromal cell | C-type lectin receptor signaling pathway | 0.5333 |
| Cortical stromal cell | Glycosaminoglycan biosynthesis | 0.7083 |
| PST cell | TNF signaling pathway | 0.4790 |
| PST cell | Phototransduction | 0.5862 |
| PST cell | Type II diabetes mellitus | 0.5319 |
| PST cell | Tight junction | 0.4588 |
| PST cell | Salivary secretion | 0.4845 |
| PSC | Biosynthesis of cofactors | 0.2532 |
| PSC | Oxidative phosphorylation | 0.1812 |
| PSC | Valine, leucine and isoleucine degradation | 0.1250 |
| PSC | Carbon metabolism | 0.2393 |
| PSC | Glyoxylate and dicarboxylate metabolism | 0.1250 |
| T cell | Arginine and proline metabolism | 0.2400 |
| T cell | Citrate cycle (TCA cycle) | 0.2000 |
| T cell | Butanoate metabolism | 0.1852 |
| T cell | Folate transport and metabolism | 0.1935 |
| T cell | Biosynthesis of amino acids | 0.2533 |
| Myofibroblast | Alanine, aspartate and glutamate metabolism | 0.2432 |
| Myofibroblast | Lipoic acid metabolism | 0.2000 |
| Myofibroblast | Glycine, serine and threonine metabolism | 0.2439 |
| Myofibroblast | Ascorbate and aldarate metabolism | 0.2258 |
| Myofibroblast | Pentose and glucuronate interconversions | 0.2222 |
| Podocyte | Ferroptosis | 0.3810 |
| Podocyte | Glycerophospholipid metabolism | 0.3301 |
| Podocyte | MAPK signaling pathway | 0.4767 |
| Podocyte | Glutathione metabolism | 0.2542 |
| Podocyte | Glutamatergic synapse | 0.5517 |
| Principal cell | Fatty acid degradation | 0.2791 |
| Principal cell | Nucleotide metabolism | 0.3059 |
| Principal cell | Pentose phosphate pathway | 0.2581 |
| Principal cell | Pyruvate metabolism | 0.2766 |
| Principal cell | Carbohydrate digestion and absorption | 0.2692 |

**Table S3 Differential expression gene from DN podocytes via the volcano plot screening.**

| **name** | **log2FC** | **Pvalue** |
| --- | --- | --- |
| MAPK14 | 8.9076 | 0.0000 |
| SLC7A11 | 8.4479 | 0.0000 |
| GPX4 | 7.8204 | 0.0000 |
| ACSL5 | 7.7805 | 0.0002 |
| CDS2 | 7.7325 | 0.0007 |
| PLD1 | 7.3058 | 0.0007 |
| DGKG | 7.1989 | 0.0018 |
| GPAT2 | 7.1423 | 0.0010 |
| ATG5 | 7.0856 | 0.0004 |
| DUSP4 | 6.8204 | 0.0005 |

**Table S4 Pathway enrichment analysis of human podocyte-specific DEGs.**

| Description | log2FoldChange | pvalue |
| --- | --- | --- |
| MAPK signaling pathway | 7.4909 | 0.0005 |
| Glutathione metabolism | 5.6727 | 0.0006 |
| Ferroptosis | 9.2744 | 0.0009 |
| Glycerophospholipid metabolism | 3.9148 | 0.0010 |
| Glutamatergic synapse | 5.0370 | 0.0012 |
| GnRH signaling pathway | 5.2356 | 0.0026 |
| GnRH secretion | 5.9927 | 0.0044 |
| Neuroactive ligand-receptor interaction | 2.6319 | 0.0046 |
| Cushing syndrome | 3.7696 | 0.0051 |
| HIF-1 signaling pathway | 4.4264 | 0.0054 |
| Pertussis | 4.9939 | 0.0083 |
| Leishmaniasis | 4.9307 | 0.0087 |
| Chemokine signaling pathway | 3.0274 | 0.0143 |
| Fatty acid biosynthesis | 10.8202 | 0.0143 |
| Apelin signaling pathway | 3.4779 | 0.0145 |
| Gap junction | 4.2340 | 0.0147 |
| Calcium signaling pathway | 2.6837 | 0.0154 |
| Phosphatidylinositol signaling system | 3.9748 | 0.0181 |
| Inflammatory mediator regulation of TRP channels | 3.9346 | 0.0187 |
| Amoebiasis | 3.7818 | 0.0213 |
| Hippo signaling pathway | 3.1013 | 0.0226 |
| Hormone signaling | 2.6680 | 0.0250 |
| Notch signaling pathway | 4.7120 | 0.0257 |
| Arachidonic acid metabolism | 4.6372 | 0.0268 |
| Toxoplasmosis | 3.4779 | 0.0280 |
| Serotonergic synapse | 3.3872 | 0.0304 |
| Wnt signaling pathway | 2.7983 | 0.0332 |
| Cytokine-cytokine receptor interaction | 2.2875 | 0.0334 |
| Renin secretion | 4.2340 | 0.0338 |
| Sphingolipid signaling pathway | 3.1928 | 0.0366 |
| Axon guidance | 2.6462 | 0.0407 |
| Relaxin signaling pathway | 2.9964 | 0.0446 |
| NOD-like receptor signaling pathway | 2.5762 | 0.0448 |
| Inositol phosphate metabolism | 3.7454 | 0.0460 |
| Dopaminergic synapse | 2.9288 | 0.0478 |

**Table S5**  **Top significantly enriched KEGG pathways in each cell type based on genes differentially expressed in db/db kidneys.**

| **Type** | **Description** | **Value** |
| --- | --- | --- |
| Renal corpusle cell | Vasopressin-regulated water reabsorption | 0.0455 |
| Renal corpusle cell | Renin-angiotensin system | 0.0541 |
| Renal corpusle cell | cAMP signaling pathway | 0.0580 |
| PT cell | Vasopressin-regulated water reabsorption | 0.0455 |
| PT cell | Protein digestion and absorption | 0.0278 |
| PT cell | Carbohydrate digestion and absorption | 0.0200 |
| GPC cell | Mineral absorption | 0.0364 |
| GPC cell | Vasopressin-regulated water reabsorption | 0.0455 |
| GPC cell | Carbon metabolism | 0.0081 |
| Loop of Henle cell | Aldosterone-regulated sodium reabsorption | 0.0263 |
| Loop of Henle cell | cAMP signaling pathway | 0.0580 |
| Loop of Henle cell | Renin-angiotensin system | 0.0541 |
| Endothelial cell | VEGF signaling pathway | 0.1034 |
| Endothelial cell | Fluid shear stress and atherosclerosis | 0.0470 |
| Endothelial cell | Leukocyte transendothelial migration | 0.0672 |
| Proliferative cell | Cell cycle | 0.0191 |
| Proliferative cell | PI3K-Akt signaling pathway | 0.0326 |
| Proliferative cell | MAPK signaling pathway | 0.0368 |
| MP cell | Fc gamma R-mediated phagocytosis | 0.0851 |
| MP cell | Chemokine signaling pathway | 0.0567 |
| MP cell | Toll-like receptor signaling pathway | 0.0288 |
| Smooth muscle cell | Vascular smooth muscle contraction | 0.0833 |
| Smooth muscle cell | cGMP-PKG signaling pathway | 0.0581 |
| Smooth muscle cell | Calcium signaling pathway | 0.0431 |
| CDC cell | Antigen processing and presentation | 0.0114 |
| CDC cell | Toll-like receptor signaling pathway | 0.0288 |
| CDC cell | NF-kappa B signaling pathway | 0.0280 |
| Podocyte | Glycerophospholipid metabolism | 0.0396 |
| Podocyte | MAPK signaling pathway | 0.0368 |
| Podocyte | Glutathione metabolism | 0.0137 |

**Table S6** **The significantly dysregulated metabolites in db/db mouse kidney section.**

| **name** | **exact_mass** | **KEGG** | **KEGG_Pathway** | **CAS** |
| --- | --- | --- | --- | --- |
| Serine | 105.04259 | C00065 | Lipid metabolism | 6898-95-9 |
| Glycerone phosphate | 169.9980252 | C00111 | Lipid metabolism | 57-04-5 |
| Glycerol 3-phosphate | 172.0136744 | C00093 | Lipid metabolism | 17989-41-2 |
| Phosphorylcholine | 184.073866 | C00588 | Lipid metabolism | 3616-04-4 |
| Cystine | 240.02385 | C00491 | Amino acid metabolism | 2079930-29-1 |
| Glutathione | 307.0838022 | C00051 | Amino acid metabolism | 70-18-8 |

**Table S7 Mantel test correlation analysis between the significant differential metabolites and clinical indices of renal function.**

| **Clinical parameters** | **Metabolites** | **correlation** | **pvalue** | **Mantels_r** | **Mantels_p** |
| --- | --- | --- | --- | --- | --- |
| Blood uric acid | Glycerol 3-phosphate | 0.1605 | 0.0160 | 0.1 - 0.2 | 0.01 - 0.05 |
| Blood uric acid | Serine | 0.1602 | 0.0470 | 0.1 - 0.2 | 0.01 - 0.05 |
| Blood uric acid | Glycerone phosphate | 0.0804 | 0.1320 | < 0.1 | >= 0.05 |
| Blood glucose | Glutathione | 0.1354 | 0.1380 | 0.1 - 0.2 | >= 0.05 |
| Serum creatinine | Glutathione | 0.0837 | 0.1530 | < 0.1 | >= 0.05 |
| Serum creatinine | Phosphorylcholine | 0.0451 | 0.2420 | < 0.1 | >= 0.05 |
| eGFR | Phosphorylcholine | 0.0360 | 0.2950 | < 0.1 | >= 0.05 |
| BUN | Cystine | 0.0392 | 0.2990 | < 0.1 | >= 0.05 |
| BUN | Glycerone phosphate | 0.0346 | 0.3040 | < 0.1 | >= 0.05 |
| Blood uric acid | Cystine | 0.0302 | 0.3210 | < 0.1 | >= 0.05 |
| eGFR | Glutathione | 0.0274 | 0.3330 | < 0.1 | >= 0.05 |
| BUN | Glycerol 3-phosphate | 0.0142 | 0.3940 | < 0.1 | >= 0.05 |
| Blood glucose | Serine | 0.0014 | 0.4150 | < 0.1 | >= 0.05 |
| Blood glucose | Glycerone phosphate | 0.0031 | 0.4170 | < 0.1 | >= 0.05 |
| Blood uric acid | Glutathione | 0.0085 | 0.4210 | < 0.1 | >= 0.05 |
| eGFR | Glycerone phosphate | 0.0037 | 0.4380 | < 0.1 | >= 0.05 |
| BUN | Serine | -0.0250 | 0.4990 | < 0.1 | >= 0.05 |
| BUN | Glutathione | -0.0205 | 0.5230 | < 0.1 | >= 0.05 |
| eGFR | Glycerol 3-phosphate | -0.0223 | 0.5370 | < 0.1 | >= 0.05 |
| Serum creatinine | Glycerone phosphate | -0.0226 | 0.5750 | < 0.1 | >= 0.05 |
| Blood glucose | Cystine | -0.0377 | 0.5800 | < 0.1 | >= 0.05 |
| Blood glucose | Glycerol 3-phosphate | -0.0286 | 0.5890 | < 0.1 | >= 0.05 |
| BUN | Phosphorylcholine | -0.0311 | 0.6500 | < 0.1 | >= 0.05 |
| Blood glucose | Phosphorylcholine | -0.0445 | 0.6510 | < 0.1 | >= 0.05 |
| eGFR | Serine | -0.0781 | 0.7130 | < 0.1 | >= 0.05 |
| Serum creatinine | Serine | -0.0733 | 0.7440 | < 0.1 | >= 0.05 |
| Blood uric acid | Phosphorylcholine | -0.0728 | 0.8890 | < 0.1 | >= 0.05 |
| Serum creatinine | Glycerol 3-phosphate | -0.0992 | 0.9130 | < 0.1 | >= 0.05 |
| eGFR | Cystine | -0.1173 | 0.9440 | < 0.1 | >= 0.05 |
| Serum creatinine | Cystine | -0.1258 | 0.9740 | < 0.1 | >= 0.05 |


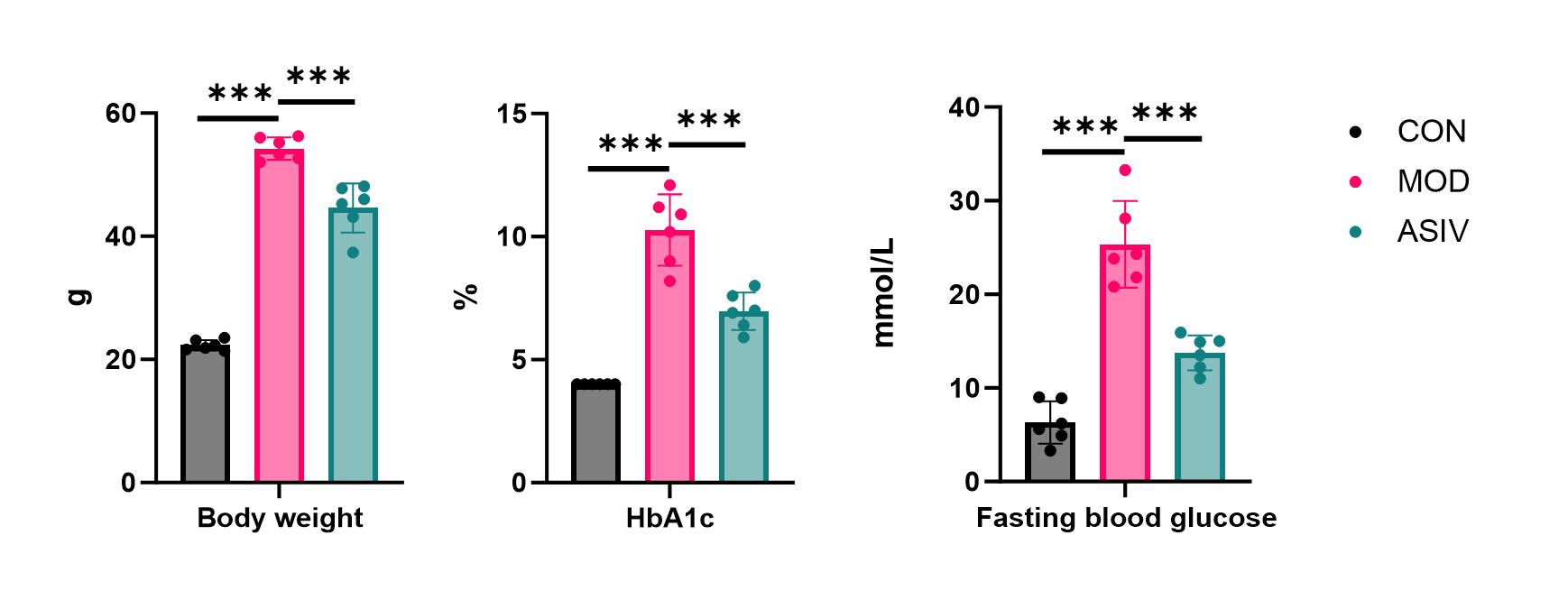


**Fig. S1 The biochemical parameters** **in experimental mice.**Data are presented as mean ± SEM (n=6 per group). Statistical analysis was performed using Student's t-test. *p < 0.05, **p < 0.01, ***p < 0.001. CON: non-diabetic db/m mice; MOD: diabetic db/db mice; ASIV: db/db mice treated with Astragaloside IV. HbA1c: glycated hemoglobin.
