# Supplementary figures and images for: MAPK14/SLC7A11/GPX4 axis dysregulation drives podocyte ferroptosis via mediating glycerophospholipid metabolism
Source: Cell Death Discov. 2026 Mar 11;12:147. doi: 10.1038/s41420-026-02990-7 (PMC13039714; doi:10.1038/s41420-026-02990-7)

**The full length uncropped original western blots used in the manuscript.**

**Fig.6i**


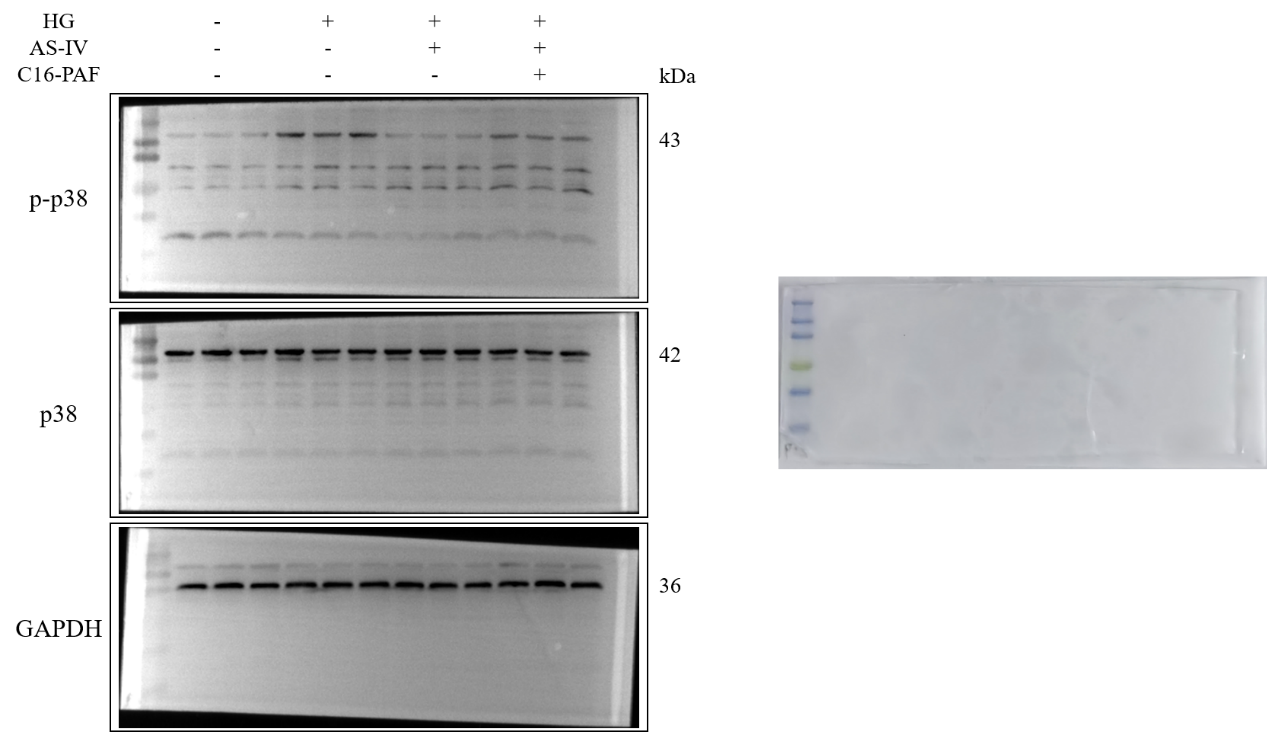


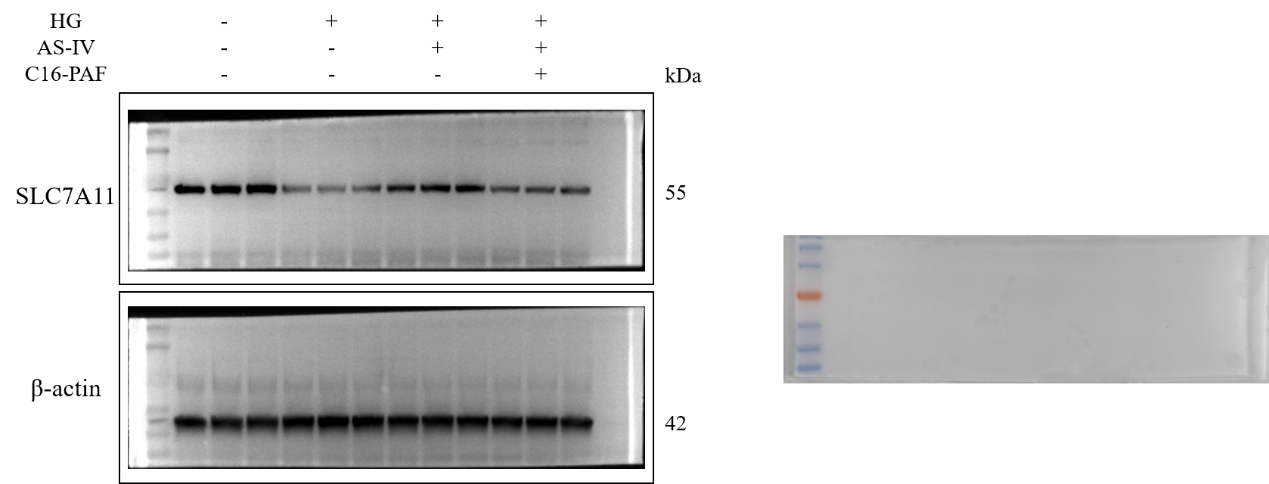


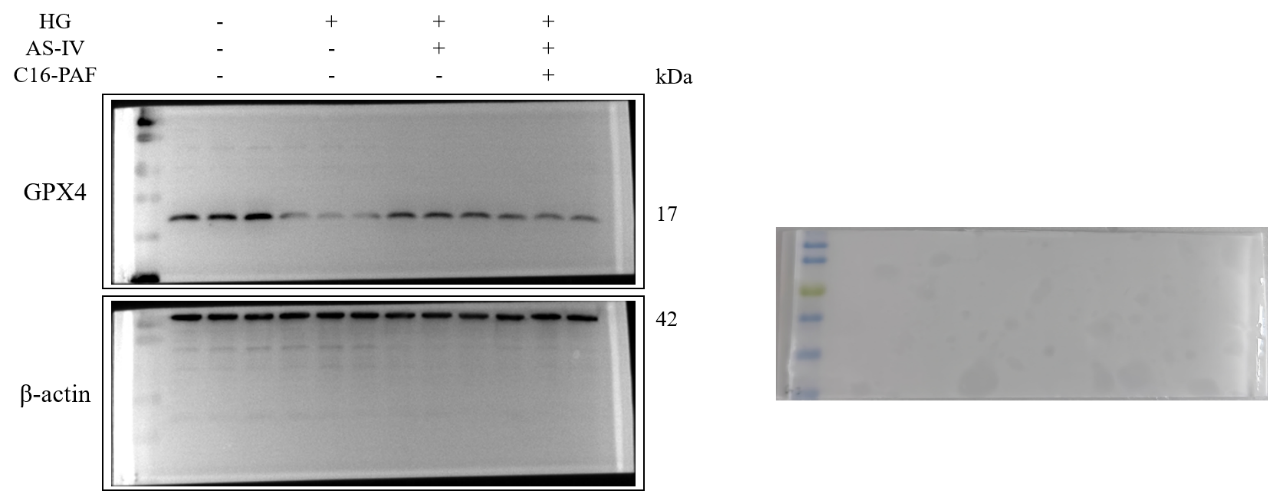


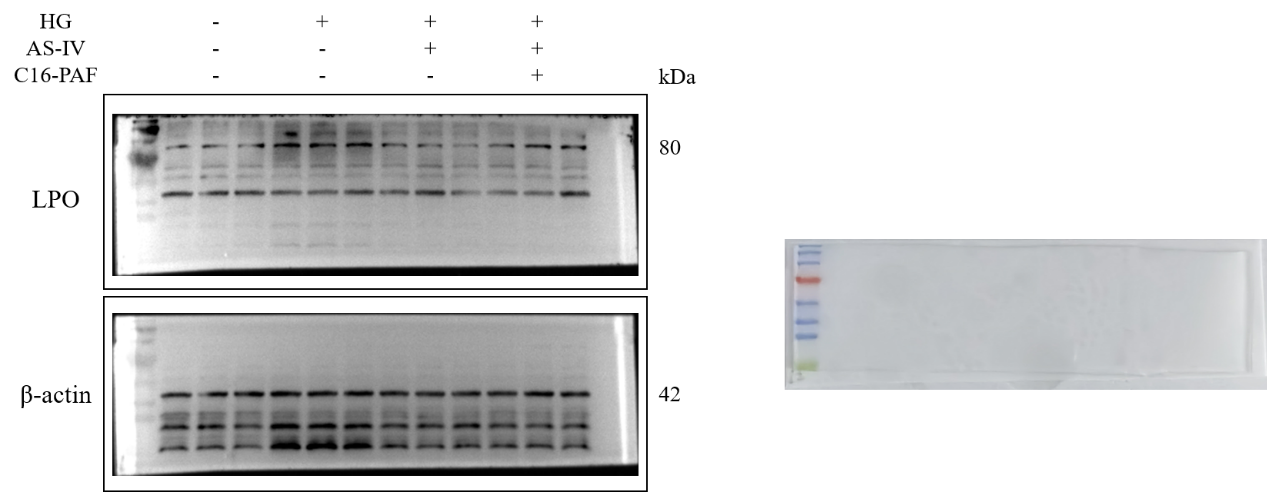

Supplement: Supplementary file 2 — Original Data Files [file 41420_2026_2990_MOESM2_ESM.docx]
